# Covering assisted intuitionistic fuzzy bi-selection technique for data reduction and its applications

**DOI:** 10.1038/s41598-024-62099-8

**Published:** 2024-06-12

**Authors:** Rajat Saini, Anoop Kumar Tiwari, Abhigyan Nath, Phool Singh, S. P. Maurya, Mohd Asif Shah

**Affiliations:** 1 Department of Mathematics, School of Basic Sciences, Central University of Haryana, Mahendergarh, 123031 India; 2 Department of Computer Science and Information Technology, Central University of Haryana, Mahendergarh, 123031 India; 3 Department of Biochemistry, Pt. Jawahar Lal Nehru Memorial Medical College, Raipur, 492001 India; 4 Department of Mathematics (SoET), Central University of Haryana, Mahendergarh, 123031 India; 5 Department of Geophysics, Institute of Science, Banaras Hindu University, Varanasi, 221005 India; 6 Department of Economics, Kebri Dehar University, 250 Kebri Dehar, Somali Ethiopia; 7 Division of Research and Development, Lovely Professional University, Phagwara, Punjab 144001 India; 8 Department of Economics, Kardan University, Parwan e Du, Kabul, 1001 Afghanistan

**Keywords:** Rough set, Granular structure, Dimensionality reduction, IF set, Instance selection, Computational biology and bioinformatics, Health care, Mathematics and computing

## Abstract

The dimension and size of data is growing rapidly with the extensive applications of computer science and lab based engineering in daily life. Due to availability of vagueness, later uncertainty, redundancy, irrelevancy, and noise, which imposes concerns in building effective learning models. Fuzzy rough set and its extensions have been applied to deal with these issues by various data reduction approaches. However, construction of a model that can cope with all these issues simultaneously is always a challenging task. None of the studies till date has addressed all these issues simultaneously. This paper investigates a method based on the notions of intuitionistic fuzzy (IF) and rough sets to avoid these obstacles simultaneously by putting forward an interesting data reduction technique. To accomplish this task, firstly, a novel IF similarity relation is addressed. Secondly, we establish an IF rough set model on the basis of this similarity relation. Thirdly, an IF granular structure is presented by using the established similarity relation and the lower approximation. Next, the mathematical theorems are used to validate the proposed notions. Then, the importance-degree of the IF granules is employed for redundant size elimination. Further, significance-degree-preserved dimensionality reduction is discussed. Hence, simultaneous instance and feature selection for large volume of high-dimensional datasets can be performed to eliminate redundancy and irrelevancy in both dimension and size, where vagueness and later uncertainty are handled with rough and IF sets respectively, whilst noise is tackled with IF granular structure. Thereafter, a comprehensive experiment is carried out over the benchmark datasets to demonstrate the effectiveness of simultaneous feature and data point selection methods. Finally, our proposed methodology aided framework is discussed to enhance the regression performance for IC50 of Antiviral Peptides.

## Introduction

The proliferation of technological breakthroughs, along with the decreasing cost of equipment, has led to a significant rise in the collection of data, both in terms of volume and variety. Consequently, this has resulted in the emergence of multiple data resources. The concept of big data encompasses a vast collection of data that is characterized by its immense volume and intricate nature. The significant increase in data has led to its widespread access across various platforms, including both free and corporate resources. The current information-centric landscape necessitates the procurement, amalgamation, and examination of large-scale data sets in order to unravel intricate medical and scientific challenges.

Reducing data is a crucial step in the preprocessing of data. The primary aim of data reduction is to decrease the size or complexity of the original dataset by retaining just the most essential and representative information. The clear benefits of data minimization revolve around mitigating time complexity and preventing excessive storage requirements. Conversely, the utilization of simplified models can be facilitated through the utilization of decreased data, hence potentially enhancing the quality of the analysis outcomes. Commonly used data reduction techniques in the field include instance selection and feature selection^1^.

These techniques can be categorized into three parts namely filter method, wrapper method, and embedded method. In filter techniques, the entire process of data reduction remains independent from different machine learning algorithms as it does not incorporate any feedback from these algorithms. In wrapper techniques, the response from the machine algorithms is incorporated to evaluate the quality of the reduced data, and hence the overall performances of these algorithms can be improved for the specific domains. Embedded techniques are used to avoid the limitations of both filter and wrapper methods. Data reduction is incorporated as the part of the machine learning algorithms in embedded techniques, hence these techniques can observe advantages of both wrapper and filter methods.

Instance selection^2^ is a crucial strategy in the field of data reduction. It involves the selection of a subset of data that effectively fulfills the original objectives of a data mining application^3^, as if the entire dataset were utilized. Through the process of instance selection^4^, it is often possible to obtain representative instances by eliminating redundant instances^5^, irrelevant instances, or errors from the original data. This approach facilitates the extraction of crucial information and enables the acquisition of high-quality results with reduced computational time, particularly when dealing with large-scale datasets. The topic of instance selection^5^ has been extensively examined across various application domains. Feature selection^6^ is a significant method for reducing data, which has been employed to enhance learning performance and reduce dimensionality^7^ by eliminating repetitive or irrelevant features.

The solution to this type of problem has been proposed by many earlier studies, although the majority of them simply conducted instance selection or feature selection^8^. Few of them simultaneously thought about choosing both instances and characteristics^9^. Additionally, the majority of earlier studies did not take into account massive data and noisy data. In order to simultaneously select features and instances in big data^10^, numerous prior researches have put forth potential solutions to this particular challenge, but with a predominant focus on instance selection or feature selection methodologies^11^. A subset of individuals contemplated the selection of both examples and qualities in tandem. Furthermore, a significant proportion of previous research failed to consider the implications of extensive and unreliable data to effectively address the challenge of selecting both features and instances in massive data^12^. In following studies, researchers have employed feature selection techniques^13^ based on genetic algorithms (GA) to address a range of difficulties. These include enhancing the accuracy of molecular illness identification^14^, providing valuable assistance to neurosurgeons during surgical procedures, among others. Particle swarm optimization (PSO) has also been employed in the feature selection process.

The work conducted by the authors in reference^15^ involved the modification of the continuous Particle Swarm Optimization(PSO) algorithm to a binary PSO algorithm for the purpose of feature selection^13^. In order to enhance the accuracy of classification, the research conducted in reference^16^ employed a combination of Particle Swarm Optimization(PSO) and Linear Discriminant Analysis(LDA)^16^. In order to mitigate the problem of premature convergence in feature selection, a Particle Swarm Optimization (PSO) algorithm incorporating three distinct methods was introduced in a previous study.

Moreover, the utilization of genetic algorithms (GA)^17^ has been widely observed in the context of instance selection^18^. Ahmad et al.^19^ employed the cooperative Particle Swarm Optimization (PSO) algorithm to choose instances in their study. Here, the enhanced immune binary particle swarm optimization (PSO) algorithm as a method for classifying time-series data was presented.

While there have been previous studies that focused on either feature selection^20^ or instance selection, only a limited number of publications, such as GA^21^, have concurrently examined tackling both feature selection^22^ and instance selection. Furthermore, a significant proportion of previous algorithms did not incorporate multiple neighborhood search methods concomitantly in order to enhance the efficiency of local solution discovery and increase the likelihood of algorithm convergence.

However, these conventional approaches usually faces the loss of information due to use of expert suggestions. Further, rough set theory^23^ was incorporated to perform simultaneous feature and instance selections^24–26^ that minimize this information loss^27^. In the rough set theory^28,29^, entire bireduct process is performed based on the information from the dataset itself^30^. Ma et al.^31^ presented a min-max feature-sample bireduct method (RBR), where unification of reduct model in rough set theory^32^ was discussed. Bi-clustering was mainly employed notion for demonstrating bireducts in rough set framework. This concept was consisted of two facets: feature subsets that are consistent with decision class and sample subset where summarisation is found to be consistent. But, discretization of data in rough set theory aided techniques^33^ again leads to information loss. This issue was further handled by fuzzy rough set theory (FRS)^34,35^ by the amalgamation of fuzzy set^35^ and rough set. FRS model is defined as an effective generalization of classical rough set model. This model is comprised of the advantages of both rough as well as fuzzy sets, which has been broadly and mainly applied to cope with the data reduction of real-valued datasets^36,37^. Jensen et al.^38^ initially employed this model to propose the idea of fuzzy rough aided dependency functions and presented an effective way to compute the reduct^39^. Chen et al. established the concept of fuzzy assisted discernibility matrix based on lower approximations and used it to compute the feature subset of a decision system^40^. Bhatt et al. proposed a squeezed computing domain to perform dimensionality reduction, which enhanced the computing efficiency in an effective way^41^. Mieszkowicz-rolka discussed a variable precision model in fuzzy rough framework to eradicate noise in the high-dimensional datasets^42^. Dai et al. introduced a dimensionality reduction technique with FRS by using a maximal discernibility pair concept^43^. Misclassification and disruption due to noise in data was handled with variable precision model by Zhao et al.^44^. In the recent years, dependency function idea in FRS framework was improved by formulating proportion of membership cardinality related to fuzzy positive region to the complete dataset with different distance functions and granular structures^34,45^. FRS model was further extended based on information from hypergraph idea to improve feature selection process in terms of time and space complexity^46^. Multi-label feature selection was presented in the recent days based on multi-correlation, robust multi-neighborhood, parameterized covering space, and mutual information^37,43^. Moreover, data reduction was also discussed by eliminating redundant instances. Jensen et al.^47^ initiated this process by improving the fuzzy rough positive region concept with threshold parameters. Optimization strategies were integrated with fuzzy rough set theory^48^ to develop simultaneous size and feature selection approaches^49,50^. These optimization concepts included sophisticated search strategies^51^, where population were considered on the basis of natural occurrences and patterns via iterations^52^. An additive dimensionality^53,54^ and example reductions^55^ was discussed by Anarki et al.^56^ through fuzzy rough set theory^57^ with a shuffled frog jumping technique. Further, frequency assisted heuristic method was employed to offer the simultaneous reduction^58^ of attribute and instances^59^ in large volume of high-dimensional data. Additionally, a harmony-search based technique by considering the threshold parameters was presented to determine the bireduct (FBR) by using the fuzzy rough sets as addressed by Mac Parthalain et al.^60^. Furthermore, Zhang et al.^61^ outlined a bi-selection model (CFBR), where both irrelevant and/or redundant samples and dimension were eliminated based on representative instances^47,62^ along with fuzzy granular structure^45^. But, fuzzy set theory^63^ was incapable in dealing with later uncertainty i.e. uncertainty due to identification. This obstacle can be easily and effectively handled by introducing the bireduct method with set theory^64^. A heuristic search aided IF rough bireduct idea was discussed by Jain et al.^65^ . This method was the extension of Parthalain et al.^60^ fuzzy rough bireduct concept, where features were selected based on the notion of dependency function and instances were eliminated by employing positive region. However, Simultaneous instance and feature elimination methods by amalgamation of IF and rough sets^24,66^ is in its nascent stage due to scarcity of works till date. Moreover, the data point and feature selection by using IF rough sets^67–69^ has not been taken care from the perspective of the significance degrees of the IF granules^70^. Further, a mining algorithm^71^ appears inefficient while coping with errors available in the entire datasets when compared to reduced dataset^72^. Additionally, noise elimination has not been considered efficaciously in the IF instance selection with IF granular structure^73^. In this paper, we consider all the above discussed limitations in the earlier models. Initially, a novel IF similarity relation is established. Then, IF lower and upper approximations are described with the help of this similarity relation. Next, a novel IF granular structure is defined on the basis of this similarity relation^74^. Moreover, relevant prepositions are proved to justify the proposed concepts. Further, significance degree of IF granule is employed to discard redundant size by removing the noise. Furthermore, significance-degree-preserved feature selection is elaborated. Thereafter, simultaneous sample and feature selection^75^ techniques are investigated over the benchmark datasets^76^ to perform a comprehensive experimental study to show the superiority of the entire method. Finally, a framework is presented based on our proposed technique to improve the regression performance for IC50 of Antiviral Peptides (AVPs). The schematic depiction of entire methodlogy is given by Figure 5. Now, we can outline the way of handling the stated issues as below:

1. This study presented IF aided ideas for simultaneous sample and dimensionality reduction methods. IF highlights both membership and non-membership grades for a particular sample, which leads to cope with uncertainty due to both judgement and identification i.e. later uncertainty.
2. Here, IF and rough sets were combined to deal with both later uncertainty and vagueness.
3. IF granular structure, covering, and proximity concepts effectively avoided noise in the information systems.
4. Representative instances were identified during elimination of redundancy in size and importance-degree-preserved reduct computation ensured the removal of redundancy and irrelevancy in conditional features.

Finally, we can highlight the major contributions of the entire work as follows:

**Major contributions of the current study**

1. This study has been initiated by establishing a novel IF similarity relation for a pair of IF data points.
2. Further, an IF rough set model is presented based on this relation by assembling IF and rough sets notions to handle uncertainty due to both judgement and identification.
3. Next, a parameterized IF granular structure is discussed based on the lower approximation to deal with noise.
4. Thereafter, covering of all the available instances is defined by using the feedback from IF granular structure.
5. Moreover, proximity of a particular instance relative to different samples available in the dataset is computed.
6. Furthermore, covering and importance degree notions are incorporated to identify representative instances.
7. This covering idea is further employed to perform the importance degree based instance elimination to remove redundant size.
8. Finally, importance-degree-preserved feature subset selection is presented to discard redundant and irrelevant dimensions.

## Theoretical background

In this segment, we present subsequent discussions of basic knowledge related to IF set, IF decision system and IF relation^77^.

### Definition 2.1

(IF set) An IF set *X* in $U^d$, where $U^d$ is well defined collection of data points/objects with

1 $$\begin{aligned} X = {\bigg <x,\rho _{X}(x),\eta _{X}(x) \bigg >}, \forall x\in U^d \end{aligned}$$

Moreover,$\rho _{X}$ and $\eta _{X}$ are depicted by mappings

2 $$\begin{aligned} \rho _{X}: U^d \rightarrow [0,1] \end{aligned}$$

and

3 $$\begin{aligned} \eta _{X}: U^d\rightarrow [0,1] \end{aligned}$$

Here, interesting condition holds, which is specified by:

$0\le \rho _{X}(x) + \eta _{X}(x)\le 1$, $\forall x$ in $U^d$. Here, the two values $\rho _{X}(x)$ and $\eta _{X}(x)$ are narrated as membership and non-membership grades respectively, $\forall x \in U^d$. Further, hesitancy degree can be narrated by $\pi _{X} \forall x \in U^d$, which is persuasively computed by the widely employed expression $\pi _{X}(x)= 1 - \rho _{X}(x) -\eta _{X}(x)$. It can be noted here that $0\le \pi _{X}(x) \le 1$, $\forall x \in U^d$. The arrayed pair $\left\{ \rho _{X},\eta _{X}\right\}$ is identified as an essential IF value.

### Definition 2.2

(IF information system) An IF information system (IFIS) is portrayed by a quadruple $( U^d, A, V_{L},L)$. Here, $V_{L}$ is comprised of all IF values, and is exemplified by $L: U^d \times A\rightarrow V_{L}$, in such a way that $IF(x, a) = <\rho _{X}(x),\eta _{X}(x)>$, $\forall x \in U^d$ and $\forall a\in A$

### Definition 2.3

(IF decision system) An IFIS $(U^d,A,V_{L},L)$ is delineated as an IF decision system iff $A= C\cup D$, where *C* is an available non-empty finite accumulation of conditional dimensions/attributes and *D* is an available non-empty accumulation of decision dimension.

### Definition 2.4

(IF relation) Let *R*(*x*, *y*) be an required IF similarity relation, where it holds/satisfies following essential conditions Reflexivity:

4 $$\begin{aligned} \rho _{R}(x,x)=1 ~and ~\eta _{R}(x,x)=0 \end{aligned}$$

Symmetry:

5 $$\begin{aligned} \rho _{R}(x,y)=\rho _{R}(y,x) and \eta _{R}(x,y)=\eta _{R}(y,x), \forall x,y \in U^d \end{aligned}$$

## Proposed work

In this segment, we incorporate the essential conditions to establish the new extensions of IF relation and IF granular structure. Further, an effective bireduct notion is discussed by presenting the instance/data point and feature/dimension selection approaches.

### Definition 3.1

(IF relation) Let $U^d$ be the assemblage of data points/tuples i.e. universe of discourse. Then, an extension IF similarity relation as $R^{c}$ over $U^d$ can be defined by the following prominent equation:

6 $$\begin{aligned} R^{c}(x_i,x_j) = \sum \limits _{i=1}^n \sum \limits _{j=1}^n \frac{(1-\frac{1}{2}(|\rho _{a}(x_{i})-\rho _{a}(x_{j})|+|\eta _{a}(x_{i})-\eta _{a}(x_{j})|))}{n} \end{aligned}$$

    $$\forall x_i,x_j \in U^d and ~\forall ~a \in B$$

### Proof

P(1). we know that value of $\rho _{a}(x_{i})$, $\rho _{a}(x_{j}$, $\eta _{a}(x_{i})$ and $\eta _{a}(x_{j})$ always lies between 0 and 1. $\Rightarrow 0 \le |\rho _{a}(x_{i})-\rho _{a}(x_{j})| \le 1$ and $0 \le |\eta _{a}(x_{i})-\eta _{a}(x_{j})| \le 1$

$$\Rightarrow 0 \le |\rho _{a}(x_{i})-\rho _{a}(x_{j})|+|\eta _{a}(x_{i})-\eta _{a}(x_{j})| \le 2$$

$$\Rightarrow 0 \le \frac{|\rho _{a}(x_{i})-\rho _{a}(x_{j})|+|\eta _{a}(x_{i})-\eta _{a}(x_{j})|}{2} \le 1$$

$$\Rightarrow 0 \le 1-\frac{|\rho _{a}(x_{i})-\rho _{a}(x_{j})|+|\eta _{a}(x_{i})-\eta _{a}(x_{j})|}{2} \le 1$$

$$\Rightarrow 0 \le \frac{1-\frac{|\rho _{a}(x_{i})-\rho _{a}(x_{j})|+|\eta _{a}(x_{i})-\eta _{a}(x_{j})|}{2}}{n} \le \frac{1}{n}$$

$$\Rightarrow \sum \limits _{i=1}^n \sum \limits _{j=1}^n 0 \le \sum \limits _{i=1}^n \sum \limits _{j=1}^n \frac{1-\frac{|\rho _{a}(x_{i})-\rho _{a}(x_{j})|+|\eta _{a}(x_{i})-\eta _{a}(x_{j})|}{2}}{n} \le \sum \limits _{i=1}^n \sum \limits _{j=1}^n \frac{1}{n}$$

$$\Rightarrow 0 \le R^{c}(x_i,x_j) \le 1$$

P(2). Check for $$x_i= x_j$$

$$R^{c}(x_i,x_i)= \sum \limits _{i=1}^n \frac{1-\frac{|\rho _{a}(x_{i})-\rho _{a}(x_{i})|+|\eta _{a}(x_{i})-\eta _{a}(x_{i})|}{2}}{n}$$

$$\Rightarrow R^{c}(x_i,x_i)= \sum \limits _{i=1}^n \frac{1-\frac{0+0}{2}}{n}$$

$$\Rightarrow R^{c}(x_i,x_i)= \sum \limits _{i=1}^n \frac{1}{n}$$

$$\Rightarrow R^{c}(x_i,x_i)=1$$ $$\Rightarrow R^{c}(x_i,x_i)$$ is reflexive.

P(3). Now, check for symmetric property $R^{c}(x_i,x_j) = \sum \limits _{i=1}^n \sum \limits _{j=1}^n \frac{(1-\frac{1}{2}(|\rho _{a}(x_{i})-\rho _{a}(x_{j})|+|\eta _{a}(x_{i})-\eta _{a}(x_{j})|))}{n}$

$$\Rightarrow R^{c}(x_i,x_j) = \sum \limits _{j=1}^n \sum \limits _{n=1}^n \frac{(1-\frac{1}{2}(|-(\rho _{a}(x_{j})-\rho _{a}(x_{i}))|+|-(\eta _{a}(x_{j})-\eta _{a}(x_{i}))|))}{n}$$

$$\Rightarrow R^{c}(x_i,x_j) = \sum \limits _{j=1}^n \sum \limits _{n=1}^n \frac{(1-\frac{1}{2}(|\rho _{a}(x_{j})-\rho _{a}(x_{i})|+|\eta _{a}(x_{j})-\eta _{a}(x_{i})|))}{n}$$

$$\Rightarrow R^{c}(x_i,x_j) = R^{c}(x_i,x_j)$$ $$\Rightarrow R^{c}(x_i,x_j)$$ is symmetric

By P(1), P(2), P(3) $R^{c}(x_i,x_j)$ is an established similarity relation. $\square$

### Example 3.2

An IF decision system is chosen, which represents judgment problem. In this dataset, the object set X={$x_1, x_2, x_3, x_4, x_5$} comprises of 5 objects. Five conditional attributes A= {$a_1, a_2, a_3, a_4, a_5$} exist in conditional attribute set and decision attribute d with two decision class {$D_1,D_2$}. Instances in decision class $D_1 = \{x_1, x_3, x_4\}$ and $D_2 = \{x_2, x_5\}$ relation $R^c$ lies between 0 and 1 for dataset (Table 1) as shown in Table 2.

**Table 1:** An information system decision table.

Objects	Attributes					
	$a_I$	$a_2$	$a_3$	$a_4$	$a_5$	*d*
$x_I$	$\langle 0.2,0.4\rangle$	$\langle 0.1,0.7\rangle$	$\langle 0.2,0.6\rangle$	$\langle 0.6,0.4\rangle$	$\langle 0.2,0.8\rangle$	1
$x_2$	$\langle 0.1,0.7\rangle$	$\langle 0.1,0.8\rangle$	$\langle 0.3,0.6\rangle$	$\langle 0.5,0.2\rangle$	$\langle 0.2,0.7\rangle$	2
$x_3$	$\langle 0.1,0.8\rangle$	$\langle 0.1,0.8\rangle$	$\langle 0.2,0.8\rangle$	$\langle 0.5,0.4\rangle$	$\langle 0.6,0.4\rangle$	1
$x_4$	$\langle 0.1,0.9\rangle$	$\langle 0.6,0.3\rangle$	$\langle 0.2,0.7\rangle$	$\langle 0.2,0.8\rangle$	$\langle 0.6,0.4\rangle$	1
$x_5$	$\langle 0.4,0.6\rangle$	$\langle 0.2,0.6\rangle$	$\langle 0.2,0.8\rangle$	$\langle 0.2,0.8\rangle$	$\langle 0.2,0.8\rangle$	2

**Table 2:** Similarity relation between objects.

Relation	Value
$R^c(x_1, x_1)$	1
$R^c(x_1, x_2)= R^c(x_2, x_1)$	0.88
$R^c(x_1, x_3)= R^c(x_3, x_1)$	0.83
$R^c(x_1, x_4)= R^c(x_4, x_1)$	0.68
$R^c(x_1, x_5)= R^c(x_5, x_1)$	0.84
$R^c(x_2, x_2)$	1
$R^c(x_2, x_3)= R^c(x_3, x_2)$	0.87
$R^c(x_2, x_4)= R^c(x_4, x_2)$	0.70
$R^c(x_2, x_5)= R^c(x_5, x_2)$	0.80
$R^c(x_3, x_3)$	1
$R^c(x_3, x_4)= R^c(x_4, x_3)$	0.81
$R^c(x_4, x_5)= R^c(x_5, x_3)$	0.77
$R^c(x_4, x_4)$	1
$R^c(x_4, x_5)= R^c(x_5, x_4)$	0.78
$R^c(x_5, x_5)$	1

### Definition 3.3

(IF rough set) Let $U^d$ be the assemblage of samples/instances and $R^{c}$ be a presented IF similarity relation (6). Now, for any given $X \subseteq U^d$, we can provide the lower and upper approximations of *X* relative to $R^{c}$, and for any $B \subseteq A$ are described respectively by the below equation:

7 $$\begin{aligned}{} & {} \underline{R^{c}_{B}}(X)= \left\{ \frac{\left( \rho _{\underline{R_{B}^{c}}_{(X)}}(x_{i}),\eta _{\underline{R^{c}_{B}}_{(X)}}(x_{i})\right) }{x_{i}}| x_{i} \in U^d \right\} \end{aligned}$$

8 $$\begin{aligned}{} & {} \overline{R^{c}_{B}}(X) =\left\{ \frac{\left( \rho _{\overline{R^{c}_{B}}_{(X)}}(x_{i}),\eta _{\overline{R^{c}_{B}}_{(X)}}(x_{i})\right) }{x_{i}}| x_{i} \in U^d\right\} \end{aligned}$$,

here, we have

9 $$\begin{aligned}{} & {} \rho _{\underline{R^{c}_{B}}{(X)}}(x_{i}) = \inf \limits _{x_{j}\in U^d} \max \left( \eta _{R^{c}}{(x_{i},x_{j})},\rho _{x}(x_{j})\right) \end{aligned}$$

10 $$\begin{aligned}{} & {} \eta _{\underline{R^{c}_{B}}{(X)}}(x_{i}) = \sup \limits _{x_{j}\in U^d} \min \left( \rho _{R^{c}}{(x_{i},x_{j})},\eta _{x}(x_{j})\right) \end{aligned}$$

11 $$\begin{aligned}{} & {} \rho _{\overline{R^{c}}_ {{B}} {(X)}}(x_{i}) = \sup \limits _{x_{j}\in U^d} \min \left( \rho _{R^{c}}{(x_{i},x_{j})},\rho _{x}(x_{j})\right) \end{aligned}$$

12 $$\begin{aligned}{} & {} \eta _{\overline{R^{c}}_{B(X)}}(x_{i})= \inf \limits _{x_{j}\in U^d} \max \left( \eta _{R^{c}}{(x_{i},x_{j})},\eta _{x}(x_{j})\right) \end{aligned}$$

Now, the available pair $\left( \rho _{\underline{R^{c}_{B}}_{(x)}}(x_{i}),\eta _{\underline{R^{c}_{B}}_{(x)}}(x_{i}) \right)$ along with $\rho _{\overline{R^{c}}_{B(x)}}(x_{i}),\eta _{\overline{R^{c}}_{B(x)}}(x_{i})$ are portrayed as an IF numbers of data point/object $x_i$ to the well depicted lower and upper approximations. If, we have $\underline{R^{c}}(x) = \overline{R^{c}}(x)$, then, we obtain $\left( \underline{ R^{c}}(x), \overline{R}(x)\right)$, which is stated as an essential IF-definable set.

### Definition 3.4

(IF granular structure) Let $(U^d,A \cup D$) be an aforesaid IF decision system with $U^d$ and a subset of conditional dimensions $B\subseteq A$. Now, the proposed IF similarity relation is employed to achieve different levels of granularity, which results in better classification performance. Here, we compute optimal feature subset based on different levels of granularity to acquire the optimized overall accuracy. The R$_c^B$ $(x_i, x_j)$ between the instances $x_i$ and $x_j$ can be used to indicate the similarity and dissimilarity between the instances by using the information from membership and non-membership values respectively. The overall impact of noise can be eliminated or minimize by putting the value of R$_c^B$ equal to zero by considering that small value is achieved due to availability of noise. This goal can be effectively achieved by constructing parameterized IF granule with $\lambda$, $\delta \in$ (0, 1] to discard noise as follows:

(i) (i) For membership grade:
  13 $$\begin{aligned}{}[x_{i}]_{\rho _{{R}_{B}^{c}}}^{\lambda _{i}}={\left\{ \begin{array}{ll} \lambda _{i},\hspace{2cm}\rho _{{R}_{B}^{c}}(x_i,x_j)\ge \lambda _{i}\\ 0,\hspace{2cm}otherwise. \end{array}\right. } \end{aligned}$$
(ii) (ii) For non-membership grade
  14 $$\begin{aligned}{}[x_{i}]_{\eta _{{R}_{B}^{c}}}^{\delta _{i}}={\left\{ \begin{array}{ll} \delta _{i},\hspace{2cm}\eta _{{R}_{B}^{c}}(x_i,x_j)\ge \delta _{i}\\ 0,\hspace{2cm}otherwise. \end{array}\right. } \end{aligned}$$
  From the above expressions, it can be easily identified that the two parameters $\lambda$ and $\delta$ successfully impact the different size of IF granules. Here, we can observe that the IF granule is based on the diversity function of IF similarity relation. Both the parameters influence the different size of the IF granule: the larger is the values of these parameters results in the larger IF granule. Hence, we can easily compute optimized feature subset and size of the large volume of high-dimensional datasets consisted of uncertainty and noise. Here, we have $\lambda _{i} ={\rho _{\underline{R^{c}_{A}}}}[x_{i}]_{D}(x_{i})>0$. Further, $[x_{i}]_{\rho _{R^{c}_{B}}}^{\lambda _{i}}$ can be exemplified as the IF granule (13) persuaded by $x_{i}$ with respect to $B \subseteq A$.

$\delta _{i} ={\eta _{\underline{R^{c}_{A}}}}[x_{i}]_{D}(x_{i})>0$. Further, $[x_{i}]_{\eta _{R^{c}_{B}}}^{\delta _{i}}$ can be exemplified as the IF granule (14) persuaded by $x_{i}$ with respect to $B \subseteq A$.

Now, the collection of the IF granules $\left[ x_{i}\right] _{\rho _{{R^{c}_{B}}}}^{\lambda _{i}}$ stimulated by all the data points/samples in $U^d$ relative to the given subset of features/attributes *B*, and is depicted by the expression $\rho GrS(U^d,B)$, where,

15 $$\begin{aligned} \rho GrS(U^d,B)=\left\{ \left[ x_{i}\right] _{\rho _{{R^{c}_{B}}}}^{\lambda {i}}:x_{i} \in U^d,i=1,2..n,B \subseteq A,\lambda _{i}={\rho _{\underline{R^{c}_{A}}}}[x_{i}]_{D}(x_{i}) \right\} \end{aligned}$$

Now, we have $\delta _{i} = {\eta _{{\underline{R^{c}_{A}}}}}[x_{i}]_{D}(x_{i})>0$. Moreover, $[x_{i}]_{\eta _{R^{c}_{B}}}^{\delta _{i}}$ can be illustrated by the IF granule stimulated by $x_{i}$ relative to the subset of dimensions *B*. Then, the set of the intutionistic fuzzy granules $\left[ x_{i}\right] _{\eta _{R^{c}_{B}}}^{\delta _{i}}$ stimulated by all the given samples/objects in $U^d$ relative to available subset of attributes/dimensions *B*, and is depicted by the expression $\eta G_{r}S(U^d,B)$, where,

16 $$\begin{aligned} \eta G_{r}S(U^d,B)=\left\{ \left[ x_{i}\right] _{\eta _{R^{c}_{B}}}^{\delta {i}}:x_{i} \in U^d, i=1,2,......n, B \subseteq A,\delta _{i}={\eta _{\underline{R^{c}_{A}}}}[x_{i}]_{D}(x_{i})\right\} \end{aligned}$$

### Example 3.5

Here, we use the example data as given in Table 1 to select the instance for different values of $\lambda _i$ and $\delta _i$

As in Tables 3, 4, and 5, various objects/instances and attributes are chosen as values of $\lambda _i$ and $\delta _i$ change.

**Table 3:** Case 1: $\lambda _i > 0.1$ and $\delta _i> 0.3$.

Objects	Attributes					
	$a_I$	$a_2$	$a_3$	$a_4$	$a_5$	*d*
$x_I$	$\langle 0.2,0.4\rangle$		$\langle 0.2,0.6\rangle$	$\langle 0.6,0.4\rangle$	$\langle 0.2,0.8\rangle$	1
$x_2$			$\langle 0.3,0.6\rangle$		$\langle 0.2,0.7\rangle$	2
$x_3$			$\langle 0.2,0.8\rangle$	$\langle 0.5,0.4\rangle$	$\langle 0.6,0.4\rangle$	1
$x_4$		$\langle 0.6,0.3\rangle$	$\langle 0.2,0.7\rangle$	$\langle 0.2,0.8\rangle$	$\langle 0.6,0.4\rangle$	1
$x_5$	$\langle 0.4,0.6\rangle$	$\langle 0.2,0.6\rangle$	$\langle 0.2,0.8\rangle$	$\langle 0.2,0.8\rangle$	$\langle 0.2,0.8\rangle$	2

**Table 4:** Case 2: $\lambda _i > 0.3$ and $\delta _i > 0.2$.

Objects	Attributes					
	$a_I$	$a_2$	$a_3$	$a_4$	$a_5$	*d*
$x_I$				$\langle 0.6,0.4\rangle$		1
$x_2$						2
$x_3$				$\langle 0.5,0.4\rangle$	$\langle 0.6,0.4\rangle$	1
$x_4$		$\langle 0.6,0.3\rangle$			$\langle 0.6,0.4\rangle$	1
$x_5$	$\langle 0.4,0.6\rangle$					2

**Table 5:** Case 3: $\lambda _i > 0.05$ and $\delta _i > 0.5$.

Objects	Attributes					
	$a_I$	$a_2$	$a_3$	$a_4$	$a_5$	*d*
$x_I$		$\langle 0.1,0.7\rangle$	$\langle 0.2,0.6\rangle$		$\langle 0.2,0.8\rangle$	1
$x_2$	$\langle 0.1,0.7\rangle$	$\langle 0.1,0.8\rangle$	$\langle 0.3,0.6\rangle$		$\langle 0.2,0.7\rangle$	2
$x_3$	$\langle 0.1,0.8\rangle$	$\langle 0.1,0.8\rangle$	$\langle 0.2,0.8\rangle$			1
$x_4$	$\langle 0.1,0.9\rangle$		$\langle 0.2,0.7\rangle$	$\langle 0.2,0.8\rangle$		1
$x_5$	$\langle 0.4,0.6\rangle$	$\langle 0.2,0.6\rangle$	$\langle 0.2,0.8\rangle$	$\langle 0.2,0.8\rangle$	$\langle 0.2,0.8\rangle$	2

To compute the importance degree of IF granule, we propose the essential definition, which can be elucidated as follows:

### Definition 3.6

Let $\left( U^d, A \cup D \right)$ be an aforementioned given IF decision system, where, $U^d$, $B\subseteq A$ and $\left[ x_{i}\right] _{\rho _{R^{c}_{B}}}^{\lambda _{i}} \in \rho GrS(U^d,B)$, for all given $x_{j} \in U^d$. Now, if $\left[ x_{i}\right] _{\rho _{R^{c}_{B}}}^{\lambda _{i}} > 0$, it can be elucidated that the available data points/instances $x_{j}$ can be covered by the discussed IF granule $\left[ x_{i}\right] _{\rho _{R^{c}_{B}}}^{\lambda _{i}}$. Here, the collection of all available instances/objects can be covered by $\left[ x_{i}\right] _{\rho _{R^{c}_{B}}}^{\lambda _{i}}$, and is depicted with

17 $$\begin{aligned} CR\left( \left[ x_{i}\right] _{\rho _{R^{c}_{B}}}^{\lambda {i}}\right) =\left\{ x_{j}: \left[ x_{i}\right] _{\rho _{R^{c}_{B}}}^{\lambda {i}}\left( x_{j}\right) >0,x_{j} \in U^d\right\} \end{aligned}$$

### Definition 3.7

Let $\left( U^d, A \cup D \right)$ be any given IF desicion system, where, $U^d$, $B\subseteq A$ along with $\left[ x_{i}\right] _{\eta _{R^{c}_{B}}}^{\delta _{i}} \in \eta GrS(U^d,B)$ for any specified tuple/object $x_{j} \in U^d$. Now, If we have $\left[ x_{i}\right] _{\eta _{R^{c}_{B}}}^{\delta _{i}} > 0$, then, we identify that the instances/examples $x_{j}$ can be covered by the IF granule $\left[ x_{i}\right] _{\eta _{R^{c}_{B}}}^{\delta _{i}}$. Here, The collection of samples/instances can be covered by the expression $\left[ x_{i}\right] _{\eta _{R^{c}_{B}}}^{\delta _{i}}$, and can be efficiently presented by the following interesting equation:

18 $$\begin{aligned} CR \left( \left[ x_{i}\right] _{\eta _{R^{c}_{B}}}^{\delta {i}}\right) = \left\{ x_{j}: \left[ x_{i}\right] _{\eta {R^{c}_{B}}}^{\delta {i}}\left( x_{j}\right) >0,x_{j} \in U^d\right\} \end{aligned}$$

Based on the Definitions 3.6 and 3.7, it can be observed that the tuple/object available in $CR \left( \left[ x_{i}\right] _{\rho _{R^{c}_{B}}}^{\lambda {i}}\right)$, additionally CR $\left( \left[ x_{i}\right] _{\eta _{R^{c}_{B}}}^{\delta {i}}\right)$ can easily be differentiated by the $\left[ x_{i}\right] _{\rho _{R^{c}_{B}}}^{\lambda {i}}$ and $\left[ x_{i}\right] _{\eta _{R^{c}_{B}}}^{\delta {i}}$ from the set of tuples/instances $U^d$. Moreover, The higher values of $| CR \left( \left[ x_{i}\right] _{\rho _{R^{c}_{B}}}^{\lambda {i}}\right) |$ and $| CR \left( \left[ x_{i}\right] _{\eta _{R^{c}_{B}}}^{\delta {i}}\right) |$ depicts that the more instances/samples can be covered by $\left[ x_{i}\right] _{\rho _{R^{c}_{B}}}^{\lambda {i}}$ and $\left[ x_{i}\right] _{\eta {R^{c}_{B}}}^{\delta _{i}}$, this clearly outlines that $\left[ x_{i}\right] _{\rho _{R^{c}_{B}}}^{\lambda {i}}$ and $\left[ x_{i}\right] _{\eta _{R^{c}_{B}}}^{\delta {i}}$ can efficiently hold more powerful discerning ability .

### Proposition 3.8

*Let* $\left( U^d, A\cup D\right)$* be a given well-described IF decision system, where* $U^d$.* If, we have an arbitrary instance/object* $x_{i}\in U^d$,* then both* $\left[ x_{i}\right] _{\rho _{R^{c}_{B}}}^{\lambda {i}} \subseteq \left[ x_{i}\right] _{\rho _{R^{c}_{H}}}^{\lambda {i}}$* and* $| CR \left( \left[ x_{i}\right] _{\rho _{R^{c}_{B}}}^{\lambda {i}}\right) | < | CR \left( \left[ x_{i}\right] _{\rho _{R^{c}_{H}}}^{\lambda {i}}\right) |$* satisfies for an available* $H\subseteq B\subseteq A$.

**Proof** If, for any given tuple/example $x_j$, which holds the condition $[x_i]_{\rho {R^{c}_{B}}}^{\lambda _i} > 0$, then we have ${\rho _{{R^{c}_{B}}}}[x_i] > \lambda _i$. Now, since, $R^{c}_{B} \subseteq R^{c}_{H}$ satisfies for an available $H \subseteq B$, and we have the criteria ${\rho _{{R^{c}_{H}}}}[x_i]> {\rho _{{R^{c}_{B}}}}[x_i] > \lambda _i$ and

$\Rightarrow {\rho _{R^{c}_{H}}} > \lambda _{i}$, then we get $\left[ x{i}\right] _{\rho _{R^{c}_{H}}}^{\lambda _{i}}(x_{j}) = \lambda _i > 0.$ Hence, we obtain $\left[ x_i\right] _{\rho _{R^{c}_{B}}}^{\lambda _{i}}(x_{j}) = \left[ x{i}\right] _{\rho _{{R^{c}_{H}}}}^{\lambda _i}(x_j)$ Now, on the contrary, for any given sample/example $x_{j}$, which holds $\left[ x_{i}\right] _{\rho _{R^{c}_{B}}}^{\lambda {i}}(x_{j})\ = 0$, therefore, we identify that ${\rho _{R^{c}_{B}}}[x_{i}](x_{j}) \le \lambda _{i}$. So, either the expression ${\rho _{{R^{c}_{F}}}}[x_{i}](x_{j})> \lambda _{i} \Rightarrow \left[ x_{i}\right] _{\rho _{R^{c}_{H}}}^{\lambda {i}}(x_{j})=\lambda _{i}>0$ or the expression ${\rho _{R^{c}_{H}}}[x_{i}](x_{j}) \le \lambda _{i} \Rightarrow \left[ x_{i}\right] _{\rho _{R^{c}_{H}}}^{\lambda {i}}(x_{j})=0$.

Therefore, we get $\left[ x_{i}\right] _{\rho _{R^{c}_{B}}}^{\lambda {i}}(x_{j})\le \left[ x_{i}\right] {\rho _{R^{c}_{H}}}(x_{j})$.

In outline, $\left[ x_{i}\right] _{\rho _{{R^{c}_{H}}}}^{\lambda {i}}(x_{j})\le \left[ x_{i}\right] {\rho _{R^{c}_{H}}}(x_{j})$ satisfies for any given $x_{j} \in U$, which results in $\left[ x_{i}\right] _{\rho _{R^{c}_{B}}}^{\lambda {i}} \subseteq \left[ x_{i}\right] _{\rho _{R^{c}_{H}}}^{\lambda {i}}$, thus obvious result can be obtained as $| {CR} \left[ x_{i}\right] _{\rho _{R^{c}_{B}}}^{\lambda _{i}}| \le$ $| {CR} \left( \left[ x_{i}\right] _{\rho _{R^{c}_{F}}}^{\lambda _{i}} \right) |$.

Further, It can be handily detected from the aforementioned Proposition 3.8 that the above discussed IF granule $\left[ x_{i}\right] _{\rho _{R^{c}_{B}}}^{\lambda _{i}}$ holds the interesting monotonicity condition. In particular, $\left[ x_{i}\right] _{\rho _{R^{c}_{B U \left\{ a \right\} }}}^{\lambda _{i}} \subseteq \left[ x_{i}\right] _{\rho _{R^{c}_{B}}}^{\lambda _{i}}$ satisfies for a given dimension $a \in A \setminus B$, which efficiently results in

$$| {CR} \left( \left[ x_{i}\right] _{\rho _{R^{c}_{B U \left\{ a \right\} }}}^{\lambda _{i}} \right) | \le | {CR} \left( \left[ x_{i}\right] _{\rho _{R^{c}_{B}}}^{\lambda _{i}} \right) |$$

### Proposition 3.9

Let $\left( U^d, A\cup D\right)$ be an available IF decision system, where $U ^d$. Now, for an existing arbitary data point $x_i \in U^d$, we have both $\left[ x_i\right] ^{\delta _i}_{\eta _{{R}_{B}^{c}} } \subseteq \left[ x_i\right] ^{\delta _i}_{\eta _{{R}_{F}^{c}} }$ as well as $|CR\left( \left[ x_i\right] _{\eta _{{R}_{B}^{c}}}\right) | \le |CR\left( \left[ x_i\right] _{\eta _{{R}_{F}^{c}}}\right) |$, which satisfies for any $H\subseteq B \subseteq A$

### Proof

It can be identified as an obvious proof similar to proposition 3.8 $\square$

### Definition 3.10

Let $\left( U^d, A\cup D\right)$ be an IF decision system, where $U^d$ is the collection of examples/instances. Now, we can illustrate the importance degree of IF granule $\left[ x_i\right] ^{\lambda _i}_{\rho _{{R}_{A}^{c}}} \in \, \rho G_{r}S\left( U^d, A\right)$ by the following expression:

19 $$\begin{aligned} H\left( \left[ x_i\right] ^{\lambda _i}_{\rho _{{R}_{A}^{c}}}\right) = \lambda _i|CR\left( \left[ x_i\right] ^{\lambda _i}_{\rho _{{R}_{A}^{c}}}\right) | \end{aligned}$$

### Definition 3.11

Let $\left( U^d, A\cup D\right)$ be an above defined IF decision system, where $U^d$ represents the given instances/samples. Then, we can depict the importance degree of IF granule $\left[ x_i\right] ^{\delta _i}_{\eta _{{R}_{A}^{c}}} \in \eta G_rS\left( U^d, A\right)$ by the below expression:

20 $$\begin{aligned} H\left( \left[ x_i\right] ^{\delta _i}_{\eta _{{R}_{A}^{c}}}\right) = \delta _i|CR\left( \left[ x_i\right] ^{\delta _i}_{\eta _{{R}_{A}^{c}}}\right) | \end{aligned}$$

Based on the above demonstrated definition 3.10 and definition 3.11, we have the importance degree of the IF granules $\left[ x_i\right] ^{\lambda _i}_{\rho _{{R}_{A}^{c}}}$ and $\left[ x_i\right] ^{\lambda _i}_{\eta _{{R}_{A}^{c}}}$, which is illustrated by two important factors; the IF lower approximation assess $\lambda _i$(for membership) and $\delta _i$(for non-membership), and the essential cardinality of coverage data points/samples set $|CR\left( \left[ x_i\right] ^{\lambda _i}_{\rho _{{R}_{A}^{c}}}\right) |$ and $|CR\left( \left[ x_i\right] ^{\delta _i}_{\eta _{{R}_{A}^{c}}}\right) |$ can be described as the more influential discriminating proficiency of the IF granule holds (17) (18). Moreover, $\lambda _i$ and $\delta _i$ is employed to contemplate the determinacy of the discerning proficiency of $\left[ x_i\right] ^{\lambda _i}_{{\rho _{{R}_{A}^{c}}}}$ and $\left[ x_i\right] ^{\delta _i}_{{\eta _{{R}_{A}^{c}}}}$. Further, if the IF granule is elicited by sample/instance $x_i$, then, the importance degree $H\left( \left[ x_i\right] ^{\lambda _i}_{\rho _{{R}_{A}^{c}}}\right)$ along with $H\left( \left[ x_i\right] ^{\delta _i}_{\eta _{{R}_{A}^{c}}}\right)$ is again illustrated as the importance degree of example/sample $x_i$ (19) (20).

Let $\left( U^d, A\cup D\right)$ be an aforementioned IF decision system and decision class $U^d/D_t = \left\{ D_t1, D_t2, D_t3, .....,D_tl\right\}$ be the important decision split. Now, we present the forward object/instance selection idea, which can be classified into two categories as follows:

(i) Initially, we contemplate to determine the noise in each available decision class $D_t\left( 1,2,3,..l\right)$, thereafter, the noise is eliminated from $D_t$. Now, we calculate $CR\left( \left[ x_i\right] ^{\lambda _i}_{\rho _{{R}_{A}^{c}}}\right)$ along with $CR\left( \left[ x_i\right] ^{\delta _i}_{\eta _{{R}_{A}^{c}}}\right)$ for any given $x_i \in D_t$. Further, the coverage potential of the noise is found to be lower. Moreover, we consider the sample $x_{i_{0}}$ along with $|CR\left( \left[ x_{i_{0}}\right] ^{\lambda _{i_{0}}}_{\rho _{{R}_{A}^{c}}}\right) | = 1$ as well as $|CR\left( \left[ x_{i_{0}}\right] ^{\delta _{i_{0}}}_{\eta _{{R}_{A}^{c}}}\right) | = 1$, which is obtained as the crucial potential possible noise provided in the IF decision system $\left( U^d, A\cup D\right)$. Subsequently, we have to further identify whether $x_{i_{0}}$ is computed as noise or not. In particular, we have to detect for the k-nearest neighbors of computed value $x_{i_{0}}$, which is delineated as the k objects that are nearest to the value $x_{i_{0}}$. Hereafter, the computed proximity of $x_{i_{0}}$ relative to the different instances given in $U^d$, which is expressed by the following equation:
  $Pro_A\left( x_{i_{0}}, x_i\right) = 1 - R^{c}_{A}\left( x_{i_{0}}, x_i\right) \left( x_i \in U^d, i \ne i_0\right)$. Moreover, we describe the cardinality of any given $x_{i} \in U$ by the below demonstration :
  21 $$\begin{aligned} K_{x_{i_{0}}} = \frac{1+ \rho _{A}\left( x_{i_{0}}\right) - \eta _{A}\left( x_{i_{0}}\right) }{2} \end{aligned}$$
  Here, $R_{A}^{c}$ is demonstrated as the IF instance selection relative to the given conditional dimensions/features set *A*. Now, the collection of the *k* -nearest neighbors with respect to $x_{i_0}$, which can illustrated as $k_{n}(x_{i_{0}})$ equal to the number of samples in $k_{n}(x_{i_{0}})$ consisted of distinct labels from the available label of $x_{i_{0}}$ is found as higher value with respect to the $k_{x_{i_{0}}}$, then, the sample/object $x_{i_{0}}$ is considered as noise. Thereafter, it should be highlighted that the existing label of an object $x_{i}$ is obtained as factual value of the decision dimension *dp*, i.e., $dp(x_{i})$. Moreover, the selection of the parameter *k* is implied in the section of the comprehensive numerical experimental study.
(ii) Now, we identify the representative samples on the basis of definition 3.4 and definition 3.6. It can be noted tha $D_{t}(t=1,2,\ldots ,l)$ portray the decision class, where the noise has been effectively removed. Here, we consider that the essential importance degree of a specified data point can also be pondered by the Definition 3.1 with respetc to each data point $x_{j} \in D_{t}$. Now, we determine $H\left( [x_{j}]_{\rho _{R^{c}_{A}}}^{\lambda _{i}}\right)$, and detect the instance $x_{j_{0}}$, which holds $\max \limits _{j \in \{1,2,\ldots ,|D_{t}|\}} H\left( [x_{j}]_{\rho _{R^{c}_{A}}}^{\lambda _{i}}\right)$. Consequently, the objects/data points covered by the $[x_{j_{0}}]_{\rho _{R^{c}_{A}}}^{\lambda _{j_{0}}}$, merely, $x_{j_{0}}$ is discarded from the $D_{t}$.

Now, for any given $x_{j_{1}} \in {CR}\left( [x_{j_{0}}]_{\rho _{R^{c}_{A}}}^{\lambda _{j_{0}}}\right)$, we assume that ${CR}\left( [x_{j_{1}}]_{\rho _{R^{c}_{A}}}^{\lambda _{j_{1}}}\right) = \phi$, which proceeds to discard $x_{j_{1}}$ from ${CR}\left( [x_{j}]_{\rho _{R^{c}_{A}}}^{\lambda _{j}}\right)$ ($x_{j} \in {CR}\left( [x_{j_{0}}]_{\rho _{R^{c}_{A}}}^{\lambda _{j_{0}}}\right)$, $j=1,2,\ldots ,|D_{t}|$).

Here, the entire depicted process is iterated till ${CR}\left( [x_{j}]_{\rho _{R^{c}_{A}}}^{\lambda _{j}}\right) = \phi$ for any given $x_{j} \in D_{t}$ ($j=1,2,\ldots ,|D_{t}|$), we get the equivalent value as $\max \limits _{j \in \{1,2,\ldots ,|D_{t}|\}} H([x_{j}]_{\rho _{R^{c}_{A}}}^{\lambda _{j}}) = 0$. Likewise, it can be inferred as $H([x_{j}]_{\eta _{R^{c}_{A}}}^{\delta _{j}}) = 0$.

From the above discussion, we can conclude that a given IF decision system $(U^d, A \cup D)$ converted into an IF decision subsystem $(U^{d*}, A \cap D)$, where $U^{d*}$ can be explained as a representative data points set.

**Algorithm 1:**
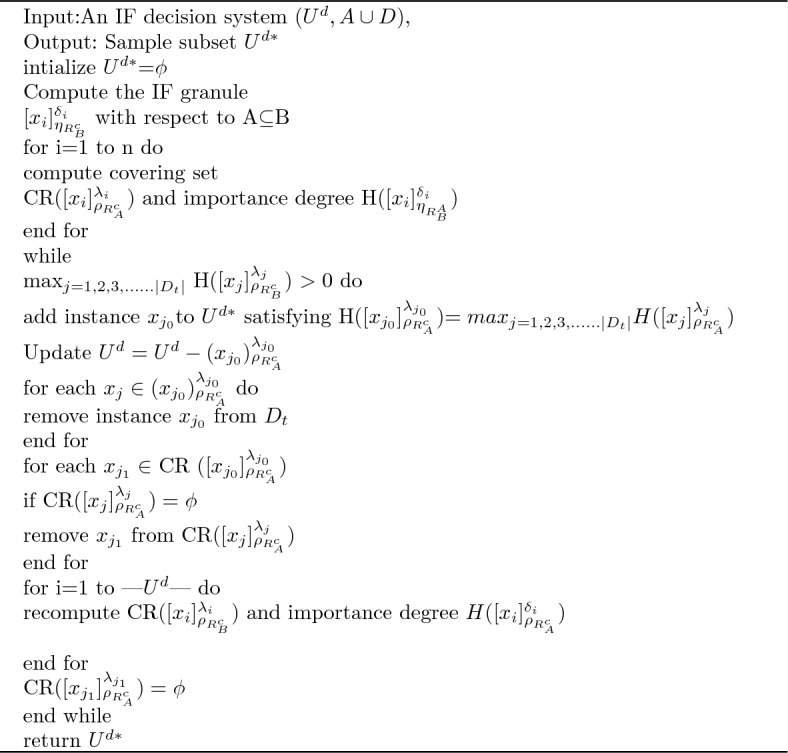
An instance elimination procedure to remove noise by identifying the representative data points

### Importance-degree-preserved IF feature selection method

In the high-dimensional datasets, redundancy and irrelevancy in dimensions can be observed frequently. Now, for above computed IF decision system $(U^{d*}, A \cup D)$, if we select the critical attribute/dimension set $A^{*}$ by preserving the essential importance degrees of the aforementioned IF granules, then we can obtain a more compact IF decision system $(U^{d*}; A^{*} \cup D)$, where the size and dimension of the datasets can be found effectively smaller. Thus, we can accomplish the objective of the interesting data reduction task. Therefore, in the current segment, importance-degree-preserved dimensionality/feature elimination for the IF decision system $(U^{d*}, A \cup D)$ is presented. Thereafter, we present the corresponding attribute/dimension selection algorithm.

Now, we will exercise all the related work based on the IF decision subsystem $(U^{d*}, A \cup D)$ to determine a new dimensionality reduction notion. Here, the collection of representative examples $U^{d*}$ can be partitioned by using the decision equivalence relation $R_{D}^{c}$ towards a category of disjoint subsets $U^{d*}/D = \left\{ {[x_{i}]_{D}^{*}, x_{i} \in U^{d*}}\right\}$, such that $[x_{i}]_{D}^{*}$ is described as the decision class of $(U^{d*}, A \cup D)$, where any given $x_{i}$ stands in $U^{d*}$. It can be outlined that, for an obtained IF decision system $(U^{d*}, A \cup D)$, we have IF lower approximation estimate of $x_{i}$ with respect to $[x_{i}]_{D}^{*}$, whcih is expressed by:

22 $$\begin{aligned} \lambda _i^* = \rho _{R_{A}^{c}} [ x_i]_D^* ( x_i) = \inf _{x_{j} \in U^d} \max \{ \eta _{R_{A}^{c}} \left( x_i,x_j \right) , \rho _{X} ( x_j) [ x_i]_D^* ( x_j ) \} \end{aligned}$$

Additionally,

23 $$\begin{aligned} \delta _i^* = \eta _{R_{A}^{c}} [ x_i]_D^* ( x_i ) = \sup _{x_{j} \in U^d} \min \{ \rho _{R_{A}^{c}} \left( x_i,x_j \right) , \eta _{X} ( x_i) [ x_i ]_D^* ( x_i ) \} \end{aligned}$$

this computation appeares to be different from the $\lambda _i, \delta _i$ for any given $(U^d, A \cup D)$ alongside $(U^{d*}, A \cup D)$, which represents the collection of all the IF granules inserted by the examples available in $U^{d*}$ related to *B*, which can be given by the following expression:

24 $$\begin{aligned}{} & {} \rho GrS(U^{d*},B)=\left\{ \left[ x_i \right] _{\rho _{R_B^{c}} }^{\lambda _i^*}, x_{i} \in U^{d*},B\subseteq A,{\lambda _{i}^{*}}=\rho _{{R}_{A}^{c}}\left[ x_{i}\right] _{D}^{*}\left( x_{i}\right) \right\} \end{aligned}$$

25 $$\begin{aligned}{} & {} \eta G_{r}S(U^{d*},B)=\left\{ \left[ x_i \right] _{\eta _{R_B^{c}} }^{\delta _i^*}, x_{i} \in U^{d*},B\subseteq A,{\delta _{i}^{*}}=\eta _{{R}_{A}^{c}}\left[ x_{i}\right] _{D}^{*} \left( x_{i}\right) \right\} \end{aligned}$$

#### Definition 3.12

Let $\left( U^{d*}, A \cup D\right)$ be an aforesaid IF decision System. Now, for any $B \subseteq A$, we can compute the improtance degree of above demonstrated IF granule as follows:

26 $$\begin{aligned} \left[ x_i \right] _{\rho _{R_B^{c}} }^{\lambda _i^*} \in \rho GrS \left( U,B \right) \ \end{aligned}$$

is

27 $$\begin{aligned} F\left[ \left[ x_i \right] _{\rho _{R_B^{c}} }^{\lambda _i^*} \right] =\frac{ \left| CR\left( \left[ x_i \right] _{\rho _{R_A^{c}} }^{\lambda _i^*} \right) \right| ^2}{ \left| CR\left( \left[ x_i \right] _{\rho _{R_B^{c}} }^{\lambda _i^*} \right) \right| }{\rho _{R_B^{c}} } \left[ x_i \right] _D^* \left( x_i \right) \end{aligned}$$

Here, we have

28 $$\begin{aligned} \rho _{\overline{{R}^{c}}_ {{B}} {(X)}}(x_{i}) = \sup \limits _{x_{j}\in U} \min \left( \rho _{R^{c}}{(x_{i},x_{j})},\rho _{x}(x_{j})\right) ) \left[ x_i \right] _{{D}}^*, \end{aligned}$$

In particular, if we have $U^{d*}=U$,then the definition 3.1 clearly indicates that the required importance degree of the aforementioned IF granule

29 $$\begin{aligned} \left[ x_i \right] _{\rho _{R_B^{c}} }^{\lambda _i} \in \rho GrS \left( U, B \right) \ \end{aligned}$$

can be given as:

30 $$\begin{aligned} F\left[ \left[ x_i \right] _{\rho _{R_B^{c}} }^{\lambda _i} \right] =\frac{ \left| CR\left( \left[ x_i \right] _{\rho _{R_A^{c}} }^{\lambda _i^*} \right) \right| ^2}{ \left| CR\left( \left[ x_i \right] _{\rho _{{R}_B^{c}} }^{\lambda _i^*} \right) \right| }{\rho _{\underline{R}_B^{c}} } \left[ x_i \right] _D^* \left( x_i \right) \end{aligned}$$.

Moreover, the calculated importance degree of IF granule

31 $$\begin{aligned} \left[ x_i \right] _{\rho _{R_B^{c}} }^{\lambda _i} \in \rho GrS \left( U^{d*}, A \cup B \right) \ \end{aligned}$$,

can be expressed by:

32 $$\begin{aligned}{} & {} F\left[ \left[ x_i \right] _{\rho _{R_A^{c}} }^{\lambda _i} \right] =\frac{ \left| CR\left( \left[ x_i \right] _{\rho _{R_A^{c}} }^{\lambda _i^*} \right) \right| ^2}{ \left| CR\left( \left[ x_i \right] _{\rho _{R_A^{c}} }^{\lambda _i^*} \right) \right| }{\rho _{R_A^{c}} } \left[ x_i \right] _D^* \left( x_i \right) \end{aligned}$$

33 $$\begin{aligned}{} & {} = \left| CR\left( \left[ x_i \right] _{\rho _{R_A^{c}} }^{\lambda _i^*} \right) \right| \, \lambda _i \end{aligned}$$

#### Definition 3.13

Let $\left( U^{d*}, A \cup D\right)$ be an obtained IF decision system. Now, for any given $B \subseteq A$, we can get the improtance degree of the IF granule as follows:

34 $$\begin{aligned} \left[ x_i \right] _{\rho _{R_B^{c}} }^{\delta _i^*} \in \rho G_r S \left( U^{d*}, A \cup B \right) \ \end{aligned}$$

can be further depicted by:

35 $$\begin{aligned}{} & {} F\left[ \left[ x_i \right] _{\eta _{R_B^{c}} }^{\delta _i^*} \right] =\frac{ \left| CR\left( \left[ x_i \right] _{\eta _{R_A^{c}} }^{\delta _i^*} \right) \right| ^2}{ \left| CR\left( \left[ x_i \right] _{\eta _{R_B^{c}} }^{\delta _i^*} \right) \right| }{\eta _{R_B^{c}} } \left[ x_i \right] _D^* \left( x_i \right) \end{aligned}$$

36 $$\begin{aligned}{} & {} \eta _{\overline{R^{c}}_{B(X)}}(x_{i})= \inf \limits _{x_{j}\in U} \max {\left( \eta _{R^{c}}{(x_{i},x_{j})},\eta _{x}(x_{j})\right) ,\left[ x_i \right] _{{D}}^*} \end{aligned}$$

In particular, if we have $U^{d*}= U$, then, the definition 3.7 clarifies that the importance degree of IF granule cab be illustrated as:

37 $$\begin{aligned} \left[ x_i \right] _{\eta _{R_B^{c}} }^{\delta _i^*} \in \eta GrS \left( U^{d*}, A \cup B \right) \ \end{aligned}$$

can be given by:

38 $$\begin{aligned} F\left[ \left[ x_i \right] _{\eta _{R_B^{c}} }^{\delta _i^*} \right] =\frac{ \left| CR\left( \left[ x_i \right] _{\eta _{R_A^{c}} }^{\delta _i^*} \right) \right| ^2}{ \left| CR\left( \left[ x_i \right] _{\eta _{R_B^{c}} }^{\delta _i^*} \right) \right| }{\eta _{R_B^{c}} } \left[ x_i \right] _D^* \left( x_i \right) \end{aligned}$$.

Thus, the required importance degree of IF granule can be demonstrated as:

39 $$\begin{aligned} \left[ x_i \right] _{\rho _{R_A^{c}} }^{\lambda _i^*} \in \rho c_r S \left( U^{d*}, A \cup B \right) \ \end{aligned}$$

can be further depicted by the expression:

40 $$\begin{aligned}{} & {} F\left[ \left[ x_i \right] _{\eta _{R_A^{c}} }^{\delta _i^*} \right] =\frac{ \left| CR\left( \left[ x_i \right] _{\eta _{R_A^{c}} }^{\delta _i^*} \right) \right| ^2}{ \left| CR\left( \left[ x_i \right] _{\eta _{R_A^{c}} }^{\delta _i^*} \right) \right| }{\eta _{R_A^{c}} } \left[ x_i \right] _D^* \left( x_i \right) \end{aligned}$$

41 $$\begin{aligned}{} & {} = \left| CR\left( \left[ x_i \right] _{\eta _{R_A^{c}} }^{\lambda _i^*} \right) \right| \, \delta _i \end{aligned}$$

#### Property 3.14

Let $(U^{d*},A \cup D)$ be an aforesaid IF decision system. Now, we have $F \left( \left[ x_{i}\right] _{\rho _{R^{c}_{E}}}^{\delta ^{*}{i}}\right) \le F \left( \left[ x_{i}\right] _{\rho _{R^{c}_{B}}}^{\delta ^{*}{i}}\right)$, which satisfies for any given $x_{i} \in U$ along with $E \subseteq B \subseteq A$.

#### Proof

For an available IF decision system $\left( U^{d*},A \cup D \right)$, we have an arbitrary example $x_i \, \in U^{d*}$, based on proposition 1, it can be obtained that $\left| CR\left( \left[ x_i \right] _{\rho _{R_E^{c}} }^{\lambda _i^*} \right) \right| \le \left| CR\left( \left[ x_i \right] _{\rho _{R_B^{c}} }^{\lambda _i^*} \right) \right|$, which satisfies for any provided $E \subseteq B$. Moreover, we have

$$\begin{aligned}{} & {} \rho _{\overline{R}_E^C} \left[ x_i \right] _D^* \left( x_i \right) = \inf _{ x_i \in U^{d*} } \max \left\{ (\eta _{R_E^C} \left( x_i,x_j \right) , \rho _x \left( x_i \right) ), \left[ x_i \right] _D^* \left( x_j \right) \right\} \le \\{} & {} \rho _{\overline{R}_B^C} \left[ x_i \right] _D^* \left( x_i \right) = \inf _{ x_i \in U^{d*} } \max \left\{ ( \eta _{R_B^C} \left( x_i,x_j \right) , \rho _x \left( x_i \right) ), \left[ x_i \right] _D^* \left( x_j \right) \right\} \end{aligned}$$,

satisfies for any provided $x_{i} \in U^{d*}$. Further, we get

$$F\left( \left[ x_{i}\right] _{\rho _{R^{c}_{E}}}^{\lambda ^{*}{i}}\right) = \frac{\left| {CR}\left( \left[ x_{i}\right] _{\rho _{R^{c}_{A}}}^{\lambda ^{*}{i}}\right) \right| ^{2}}{\left| {CR}\left( \left[ x_{i}\right] _{\rho _{R^{c}_{E}}}^{\lambda ^{*}{i}}\right) \right| } {\rho _{\underline{{R^{c}_{E}}}}\left[ {x_{i}}\right] ^{*}_{D}(x_{i})} \le \frac{\left| {CR}\left( \left[ x_{i}\right] _{\rho _{R^{c}_{A}}}^{\lambda ^{*}{i}}\right) \right| ^{2}}{\left| {CR}\left( \left[ x_{i}\right] _{\rho _{R^{c}_{B}}}^{\lambda ^{*}{i}}\right) \right| } \rho _{R^{c}_{B}}\left[ {x_{i}}\right] ^{*}_{D}(x_{i}) = F \left( \left[ x_{i}\right] _{\rho _{R^{c}_{B}}}^{\lambda ^{*}{i}}\right)$$ $$\square$$

we are aware of importance degree of IF granular structure $[x_{i}]_{\rho _{R^{c}_{E}}}^{\delta ^{*}{i}}$ established by two determining factor: the IF lower approximation $\lambda _{i}$ and the cardinality of the coverage object set $|{CR}( \left[ x_{i}\right] _{\rho _{R^{c}_{B}}}^{\lambda ^{*}{i}}|$ and in summary, importance degree of IF satisfies monotonicity.

#### Property 3.15

Let $(U^{d*},A \cup D)$ be an aforesaid IF decision system. Now, $F \left( \left[ x_{i}\right] _{\eta _{R^{c}_{E}}}^{\delta ^{*}{i}}\right) \le F \left( \left[ x_{i}\right] _{\eta _{R^{c}_{B}}}^{\delta ^{*}{i}}\right)$, satisfies for any given $x_{i} \in U$ along with the available $E \subseteq B \subseteq A$.

#### Proof

The proof can be given in the same way as property 3.14 $\square$

#### Definition 3.16

Let $(U^{d*}, A \cup D)$ be an aforementioned IF decision system. Now, a computed $B \subseteq A$ can be described as importance degree preserved aided consistent collection in the computed IF decision system $(U^{d*}, A \cup D)$. Now, if $F\left( \left[ x_{i}\right] _{\rho _{R^{c}_{B}}}^{\lambda {i}} \right) = F\left( \left[ x_{i}\right] _{\rho _{R^{c}_{A}}}^{\lambda {i}} \right)$, satisfies for an available $x_{i} \in U^{d*}$, *B* can be depicted as importance degree preserved feature subset of $\left( U^{d*}, A \cup D \right)$. Further, if *B* is calculated as importance degree preserved assisted consistent collection in the calculated IF decision system $\left( U^{d*}, A \cup D \right)$. Thereafter, there holds $x_{i_{0}} \in U^{d*}$ in such a way that $F\left( \left[ x_{i_{0}}\right] ^{\lambda ^{*}_{i_{0}}}_{\rho _{R^{c}_{B}}-\left\{ a\right\} }\right) < F\left( \left[ x_{i_{0}}\right] ^{\lambda ^{*}_{i_{0}}}_{\rho _{R^{c}_{B}}}\right)$, which satisfies for a given $a \in B$

#### Definition 3.17

Let $\left( U^{d*}, A \cup D \right)$ be an aforesaid IF decision system. Now, $B \subseteq A$ can be explained as an importance degree preserved assisted consistent collection of the calculated IF decision system $\left( U^{d*}, A\cup D \right)$. Further, if $F([x_{i}]_{\eta _{R^{c}_{B}}}^{\delta _{i}^{*}}) = F([x_{i}]_{\eta _{R^{c}_{A}}}^{\delta _{i}^{*}})$, satisfies for a provided $x_{i_{0}} \in U$. Moreover, *B* can be illustrated as importance degree preserved feature subset of a computed IF decision system $(U^{d*}, A \cup D)$. Furthermore, if *B* is obtained as an importance degree preserved aided consistent collection of $(U^{d*}, A \cup D)$, where $x_{i_{0}} \in U^{d*}$ holds in such a way that $F([x_{i_{0}}]_{\eta _{R^{c}_{B-\left\{ a \right\} }}}^{\delta _{i_{0}}^{*}}) < F([x_{i_{0}}]_{\eta _{R^{c}_{B}}}^{\delta _{i_{0}}^{*}})$, which satisfies for a given $a \in B$.

#### Definition 3.18

Let $(U^{d*}, A \cup D)$ be a computed IF decision system. Now, if $B \subseteq A$, then the sum of computed importance degrees for all the calculated objects/samples in $U^{d*}$ relative to *B*, which can be indicated by the following appealing equation:

42 $$\begin{aligned} \rho \Psi _{U^{d*}}(B) = \sum \limits _{x_{i}\in U^{d*}} F([x_{i}]_{\rho _{R^{c}_{B}}}^{\lambda _{i}^{*}}) \end{aligned}$$

#### Definition 3.19

Let $(U^{d*}, A \cup D)$ be a calculated IF decision system. Now, if $B \subseteq A$, then the addition of the aforesaid importance degree for all the examples in representative collection of samples $U^{d*}$ relative to feature subset *B*, which is given by the expression

43 $$\begin{aligned} \eta \Psi _{U^{d*}}(B) = \sum \limits _{x_{i}\in U^{d*}} F([x_{i}]_{\eta _{R^{c}_{B}}}^{\delta _{i}^{*}}) \end{aligned}$$

#### Property 3.20

Let $(U^{d*}, A \cup D)$ be aforementioned IF decision system. Then, we have $\rho \Psi _{U^{d*}},\left( M\right) \le \rho \Psi _{U^{d*}}\left( B\right)$, which satisfies for any given $M\subseteq B\subseteq A$

#### Proof

On the basis of Definition 3.16, it can be easily implied with the help of Property 3.14. $\square$

#### Property 3.21

Let $(U^{d*}, A \cup D)$ be aforesaid IF decision system. Now, $\eta \Psi _{U^{d*}}\left( M\right) < \eta \Psi _{U^{d*}},\left( B\right)$, satisfies for any provided $M\subseteq B\subseteq A$

#### Proof

On the basis of Definition 3.17, it can be easily implied with the help of Property 3.15. $\square$

#### Theorem 3.22

*Let* $(U^{d*}, A \cup D)$* be a computed IF decision system. Now, if* $B \subseteq A$* is a calculated importance degree preserved feature subset with respect to* $(U^{d*}, A \cup D)$* if and only if* $\rho \Psi _{U^{d*}}(B) = \rho \Psi _{U^{d*}}(A)$* along with* $\rho \Psi _{U^{d*}}(B - \left\{ a \right\} ) < \rho \Psi _{U^{d*}}(B)$* based on a given* $a \in B$.

#### Proof

Let $B\subseteq A$ is a calculated importance degree preserved aided consistent collection of the $(U^{d*}, A \cup D)$ $\Longleftrightarrow$ $F([x_{i}]_{\rho _{R^{c}_{B}}}^{\lambda _{i}^{*}})= F([x_{i}]_{\rho _{R^{c}_{A}}}^{\lambda _{i}^{*}})$ for each $x_{i} \in U^{d*}$ $\Longleftrightarrow$ $\sum \limits _{x_{i}\in U^{d*}} F([x_{i}]_{\rho _{R^{c}_{B}}}^{\lambda _{i}^{*}}) = \sum \limits _{x_{i}\in U^{d*}} F([x_{i}]_{\rho _{R^{c}_{A}}}^{\lambda _{i}^{*}})$ $\Longleftrightarrow$ $\rho \Psi _{U^{d*}}(B) = \rho \Psi _{U^{d*}}(A)$ by using the Definition 3.6.

Moreover, if we have $x_{i_{0}} \in U^{d*}$ in such a way that $F([x_{i_{0}}]_{\rho _{R^{c}_{B-\left\{ a \right\} }}}^{\lambda _{i_{0}}^{*}}) < F([x_{i_{0}}]_{\rho _{R^{c}_{B}}}^{\lambda _{i_{0}}^{*}})$, which effectively satisfies for any provided $a \in B$, $\Longleftrightarrow \sum \limits _{x_{i}\in U^{d*}} H([x_{i}]_{\rho _{R^{c}_{B-\left\{ a\right\} }}}^{\lambda _{i}^{*}}) < \sum \limits _{x_{i}\in U^{d*}} F([x_{b}]_{\rho _{R^{c}_{B}}}^{\lambda _{i}^{*}})$, which efficiently satisfies for an available $a \in B$, $\Longleftrightarrow \rho \Psi _{U^{d*}},\left( B-\left\{ a \right\} \right) < \rho \Psi _{U^{d*}}\left( B\right)$ for any $a \in B$.

Further, it can be observed based on the Definition 3.16 that *B* can be identified that an importance degree preserved feature subset iff $\rho \Psi _{U^{d*}}(B) = \rho \Psi _{U^{d*}}(A)$ and $\rho \Psi _{U^{d*}}(B-\{a\}) < \rho \Psi _{U^{d*}}(B)$, for all $a \in B$. $\square$

Let $\left( U^*, X \cup D\right)$ be an IF decision system with $X=\left\{ x_1, x_2, \ldots , x_j\right\}$, and $U^*$ be a representative instance set. Assume that the attributes $x_{i_1}, x_{i_2}, \ldots$ are added into the null set one by one according to the magnitude of their respective obtained $\rho \Psi _{U^*}$. The process continues until there exists some $t \in \{1,2, \ldots , m\}$ such that $\rho \Psi _{U^*}\left( \left\{ x_{i_1}, x_{i_2}, \ldots , x_{i_t}\right\} \right) =\rho \Psi _{U^*}(X)$. It is obtained from above Property that $\rho \Psi _{U^*}\left( \left\{ x_{i_1}\right\} \right) \leqslant \rho \Psi _{U^*}\left( \left\{ x_{i_1}, x_{i_2}\right\} \right) \leqslant \ldots \leqslant$ $\rho \Psi _{U^*}\left( \left\{ x_{i_1}, x_{i_2}, \ldots , x_{i_m}\right\} \right) =\rho \Psi _{U^*}(A)$.

#### Theorem 3.23

*Let* $(U^{d*}, A \cup D)$* be a calculated IF decision system. Now*, $B \subseteq A$* can be depicted as an importance degree preserved feature subset of an available* $(U^{d*}, A \cup D)$* iff* $\eta \Psi _{U^{d*}}(B) = \eta \Psi _{U^{d*}}(A)$* and* $\eta \Psi _{U^{d*}}(B - \left\{ a \right\} ) < \eta \Psi _{U^{d*}}(B)$* for any* $a \in B$.

#### Proof

The proof can be given similar to Theorem 3.22 $\square$

**Algorithm 2:**
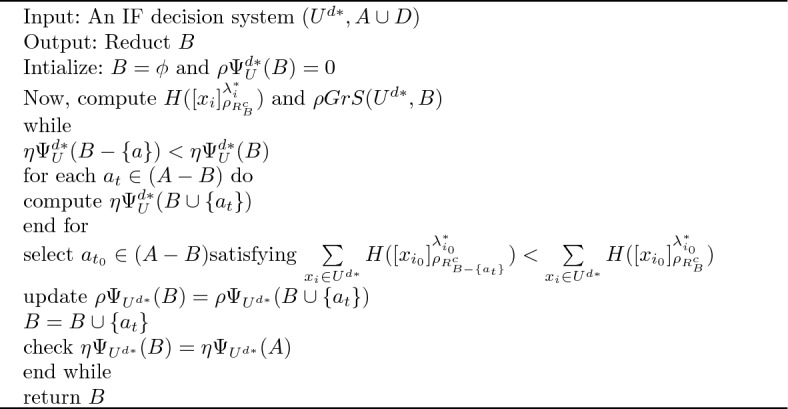
Feature subset selection procedure based on importance degree preserved notion

## Experimentation

In this segment, we have conducted thorough experiments, which demonstrates the overview and experimental verification of our illustrated methods. Here, we perform the classification performance for the effectiveness of the proposed models. Moreover, data reduction has been employed to improve the regression performance for the values of AVPs. These experiments are conducted by using Matlab 2023 and WEKA 3.8^78^.

### Performance evaluation parameters

Machine learning techniques have been evaluated with both threshold dependent and threshold independent evaluation metrics. These metrics can be calculated from the widely described quadrants of the confusion matrix, which can be outlined as True Positive (TRP), True Negative (TRN), False Positive (FLP), and False Negative (FLN). Based on these parameters, following performance evaluation metrics have been considered for addressing the overall results after data reduction: Sensitivity: This parameter depicts the average percentage of correctly predicted positive class value, which is determined by:

44 $$\begin{aligned} Sensitivity=\frac{TRP}{((TRP+FLN) )}\times 100 \end{aligned}$$

Specificity: This parameter offers the overall percentage of correctly predicted negative class values, which is estimated by:

45 $$\begin{aligned} Specificity=\frac{TRN}{((TRN+FLP) )}\times 100 \end{aligned}$$

Accuracy: This metric explains the average percentage of correctly classified positive and negative class values, which can be assessed as follows:

46 $$\begin{aligned} Accuracy=\frac{(TRP+TRN)}{(TRP+FLP+TRN+FLN)} \times 100 \end{aligned}$$

AUC: It validates the calculated value of area under the receiver operating characteristic curve (ROC). If the obtained value of AUC is nearer to 1, then, the predictor is found to be as the better performing one. MCC: Mathew’s correlation coefficient is widely and expansively used performance metric for determining better binary classification task. A MCC value calculated as 1 depicts that the classifier is the best performer when compared to others, which can be defined by using the well-known following equation:

47 $$\begin{aligned} MCC=\frac{(TRP \times TRN-FLP \times FLN)}{\sqrt{((TRP+FLP)(TRP+FLN)(TRN+FLP)(TRN+FLN)}} \end{aligned}$$

Moreover, the regression analysis is evaluated by using the broadly discussed correlation coefficient (CC) and mean absolute errors (MAE). If the values of correlation coefficient is higher and mean absolute error is less, then the regression performance is outlined as the better one.

Precision: This evaluation parameter can be calculated by the following equation:

48 $$\begin{aligned} Precision = \frac{TRP}{TRP+FLP} \end{aligned}$$

Recall: This evaluation parameter can be calculated as follows:

49 $$\begin{aligned} Recall = \frac{TRP}{TRP+FLN} \end{aligned}$$

F-measure: This evaluation metric can be computed by the following expression:

50 $$\begin{aligned} F-measure = \frac{2 \times Precision \times Recall }{Precision + Recall} \end{aligned}$$

### Result and discussion

**Figure 1:**
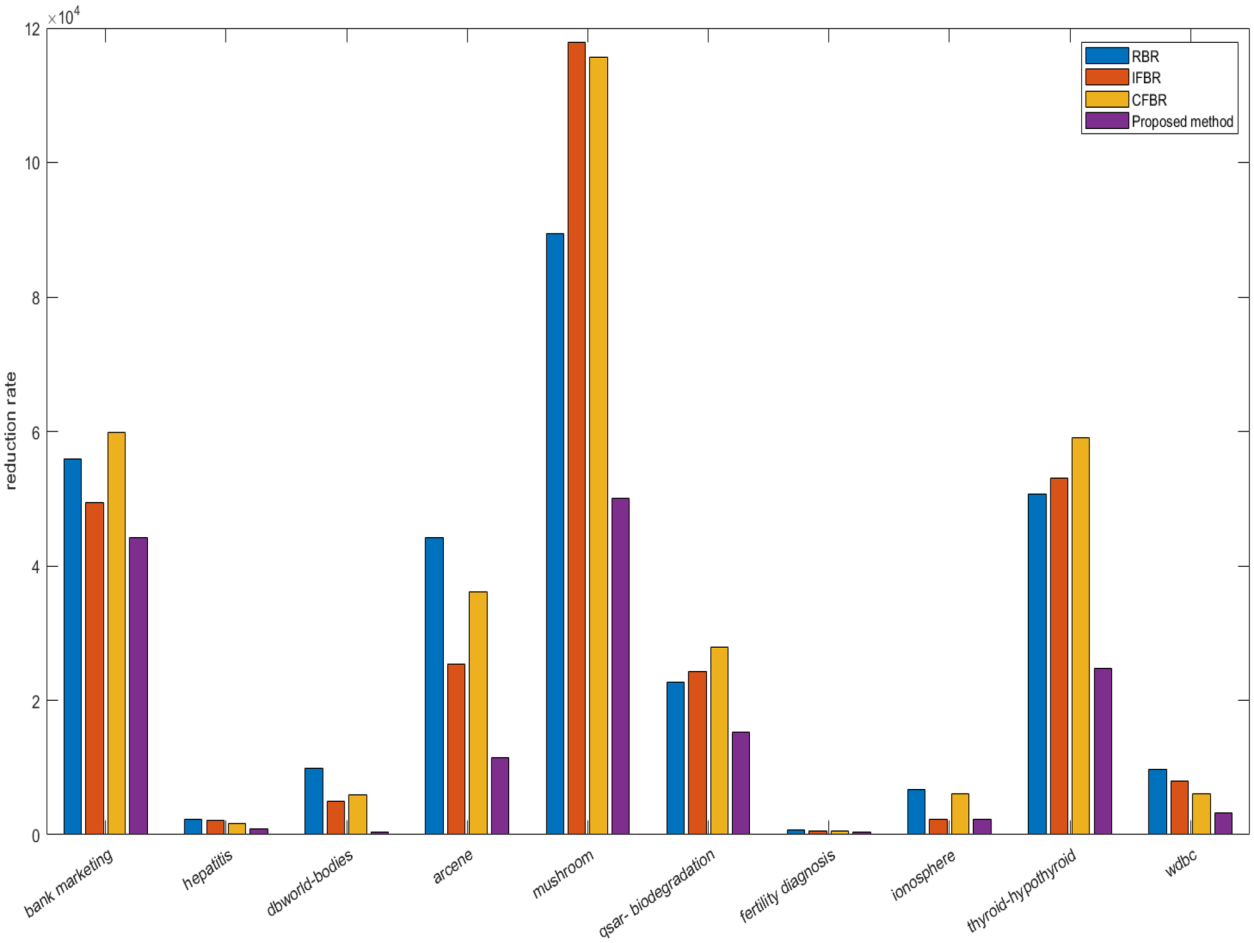
Comparison of overall reduction for different datasets by previous and proposed methods.

**Figure 2:**
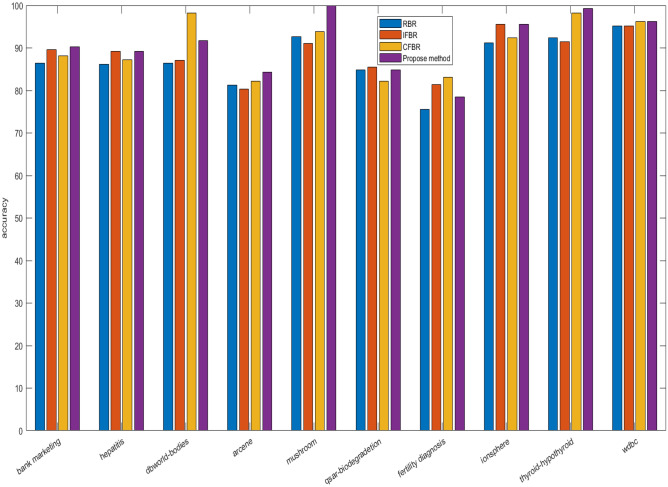
Comparison of average accuracies by JRip for different reduced datasets as produced by existing and proposed methods.

**Figure 3:**
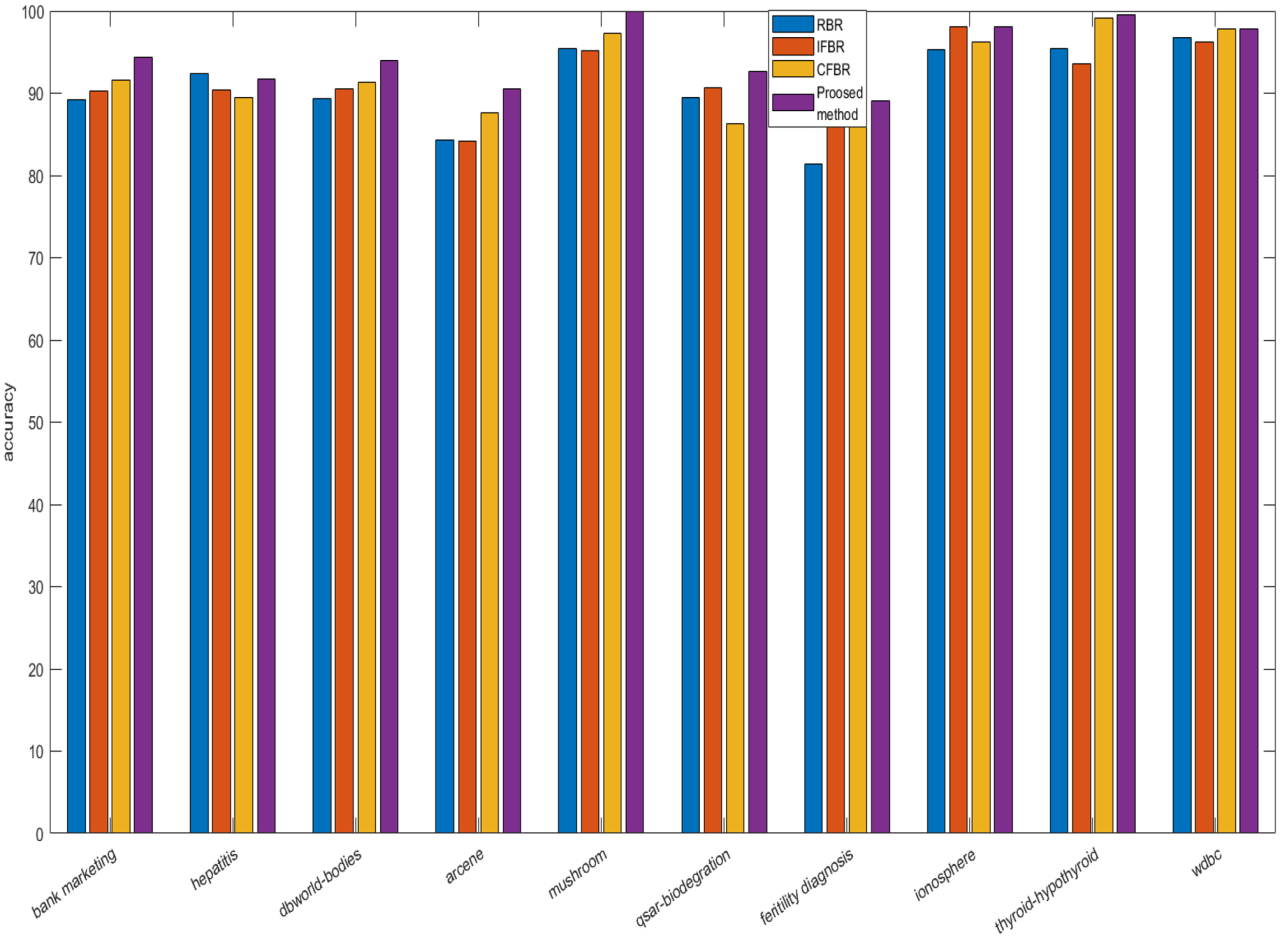
Comparison of average accuracies by RF for different reduced datasets as produced by existing and proposed methods.

We have evaluated previous as well as proposed method based on ten benchmark datasets, which are taken from UCI repository and their characteristics are depicted in Table 6. In this study, different experiments are carried out on the basis of both 10-fold cross validation and 70:30 percentage split techniques. Initially, the datasets are converted into IF information systems by employing Tan et al.^70^ method. Lower approximation is computed based on the proposed relation. Importance degree of IF granule is computed based on lower approximation. Then, redundant instances are eliminated by using this importance degree. Further, feature selection is applied based on importance degree preserved concept as discussed in “Proposed work”. The reduction rate by earlier and current approaches are reported in Table 6. Instances removed by RBR is higher in number rather than our technique for bank-marketing, dbworld-bodies, Arcene, qsar-biodegradation, and thyroid-hypothyroid, but features are discarded relatively less in number for all the datasets. RBR reduces the instances from 59 to 7456, and features from 7 to 243 as mentioned in Table 6. IFBR obliterates more samples for dbworld-bodies, Arcene, and qsar-biodegradation than proposed approach, but removal of features is found less for all the datasets. For IFBR, instances and attributes varies from 46 to 7365 and from 6 to 154 respectively. Next, CFBR eradicates more number of data points for hepatitis, dbworld-bodies, Arcene, qsar-biodegradation, and Wdbc, but dimensionality is obtained higher in count for all the datasets yet again. For CFBR, samples and dimensions vary from 41 to 7711 and from 7 to 203 respectively. Our proposed method produced the reduced datasets, where size and dimension changes from 61 to 7149 and from 5 to 60. Now, we can conclude that overall dimension in terms of both samples and features are highly reduced by our method when compared to previous techniques, as displayed by Fig. 1. Now, two broadly illustrated classifiers namely JRip and Random Forest (RF) are applied to evaluate the performances over the reduced datasets produced by RBR, IFBR, CFBR, and our technique based on overall accuracies and standard deviation. The proposed method produces better results except in the few cases for the datasets namely hepatitis, qsar-biodegradation, fertility diagnosis, ionosphere, and Wdbc. IFBR generates more effective results for hepatitis, qsar-biodegradation, and fertility diagnosis by JRip with accuracies and standard deviation of 89.24$\%$, 85.54$\%$, 81.41$\%$ and 8.31, 2.54, 10.18 respectively. Further, this method gives the equal results with respect to our method by both JRip and RF for ionosphere dataset with average accuracies and standard deviation of 95.55$\%$, 98.12$\%$ and 3.78, 2.67 respectively. RBR yields in better performance for hepatitis dataset by RF 92.58$\%$ and 5.98 and equivalent measure for qsar-biodegradation by JRip with accuracies and standard deviation of 87.80$\%$ and 3.87. CFBR presents more efficacious evaluation measure by JRip for fertility diagnosis with average accuracies and standard deviation of 83.17 $\%$ and 9.99, whilst equivalent metrics values for Wdbc by both JRip and RF with average accuracies and standard deviation of 96.20 $\%$, 97.86 $\%$ and 2.47, 2.67 respectively. From Table 7, it is obvious that our method is performing much better when compared to the existing approaches, and the best results are achieved for mushroom dataset by RF with average accuracy 99.99$\%$ and standard deviation 0.04 $\%$. The increase in overall accuracies and decrease in corresponding standard deviation clearly depicts that the proposed methodology improve the prediction performance of the learning models irrespective of reduction rate. Results from Table 6 and Table 7 highlights that our proposed methodology produces the most informative size and dimensions when compared to existing approaches in the literature. These results can be effectively visualized by the given Figs. 2 and 3. The average accuracies produced by different machine learning algorithms have been usually obtained as effective values based on our proposed when compared to the pervious approaches are obtained higher irrespective of reduced number of dimensions and size. Now, we apply the widely and effectively explained statistical test namely Freidman test^79^ and Bonferoni Dunn test^80^to evince the significance of the current approach. Freidman test is exercised to demonstrate multiple hypotheses testing by using F-statistics, which can be illustrated by the following equations:

51 $$\begin{aligned} F=\frac{(m-1) Z^2}{m(l-1)-Z^2} \end{aligned}$$

and

52 $$\begin{aligned} Z^2=\frac{12 m}{l(l+1)}\left( \sum _{i=1}^l r_i^2-\frac{l(l+1)^2}{4}\right) \end{aligned}$$

Here, m and *l* indicate the count of employed datasets and algorithms, and r$_i$ depict the calculated value of average rank of ith algorithm, which is computed from all the information systems. Further, if the F-statistics with respect to the distribution of Freidman is obtained greater than existing critical value (Fcrit) at (*l*-1) and (*l*-1)(m-1) degrees of freedom, then the null hypothesis which explains that all algorithms are equivalent in the context of average classification accuracy is declared as rejected. In this scenario, Bonferroni Dunn is applied to identify that a particular algorithm is significantly divergent from the presented method. Two methods can be said as significantly divergent at $\alpha \%$ significance level if and only if the distance available between their $r_i$ is larger than a critical distance (Cd$_\alpha$), which can be outlined by the following expression:

53 $$\begin{aligned} Cd_\alpha = q_\alpha \sqrt{\frac{l(l+1)}{6m}} \end{aligned}$$

In this expression, q$_\alpha$ is a tabular value at $\alpha \%$ significance level.

The test results are recorded in Table 7, where individual rank is given in the superscript of respective results. In this study, the average rank values by our proposed method for all the ten reduced datasets based on both machine learning algorithms namely JRip and RF are computed as 1.55 and 1.20 as reported in Table 7. These values are lower when compared to the average rank values based on other existing techniques, which clearly indicates the effectiveness of the proposed method. Moreover, F-statistics values by using both the learning algorithms based on our proposed technique are calculated as 5.68 and 9.29, whilst F-tabular value at 5$\%$ level of significance is F(3,27) $=$ 2.960, which exhibits that the null hypothesis is rejected on the basis of Freidman test. Hence, all the four algorithms are obtained as statistically different. Further, by using Bonferoni Dunn test, it can be concluded that our proposed technique is statistically superior to earlier algorithms for both the learning algorithms at 5$\%$ significance level.

**Table 6:** Benchmark datasets characteristics and their reduction.

Dataset	Instances	Attributes	Reduction							
			RBR		IFBR		CFBR		Proposed method	
			Instances	Attributes	Instances	Attributes	Instances	Attributes	Instances	Attributes
Bank marketing	4521	16	3987	14	4121	12	4278	14	4023	11
Hepatitis	155	19	151	15	147	14	137	12	147	6
Dburorld-bodies	64	4702	59	168	46	109	41	144	61	7
Arcene	200	10000	182	243	165	154	178	203	190	60
Mushroom	8124	21	7456	12	7365	16	7711	15	7149	7
qsar-biodegradation	1055	41	987	23	898	27	902	31	1012	15
Fertility diagnosis	100	9	91	7	95	6	89	7	86	5
Ionosphere	351	33	323	21	319	7	334	18	319	7
Thyroid-hypothyroid	3163	25	2977	17	3124	14	3107	19	3099	8
Wdbc	569	21	543	18	533	15	509	12	529	6

**Table 7:** Classification accuracy with standard deviation comparison with other state of art bireduct algorithms.

Dataset	Classifier	RBR	IFBR	CFBR	Proposed method
Bank	JRip	86.44$\pm 3.22^4$	89.65$\pm 2.31^2$	88.12$\pm 2.32^3$	90.27$\pm 1.15^1$
Marketing	RF	89.23$\pm 3.25^4$	90.22$\pm 3.01^3$	91.59$\pm 2.65^2$	94.37$\pm 1.01^1$
Hepatitis	JRip	86.19$\pm 6.52^4$	89.24$\pm 8.31^1$	87.23$\pm 8.11^3$	89.14$\pm 7.67^2$
	RF	92.34$\pm 5.98^1$	90.34$\pm 7.22^3$	89.44$\pm 7.97^4$	91.86$\pm 7.22^2$
dbworld	JRip	86.41$\pm 10.24^4$	87.12$\pm 11.21^3$	89.21$\pm 12.56^2$	91.74$\pm 10.08^1$
Bodies	RF	89.32$\pm 9.65^4$	90.54$\pm 9.98^3$	91.32$\pm 10.87^2$	93.93$\pm 8.32^1$
Arcene	JRip	81.27$\pm 11.12^3$	80.31$\pm 8.78^4$	82.21$\pm 9.95^2$	84.32$\pm 9.07^1$
	RF	84.27$\pm 9.32^3$	84.23$\pm 7.98^4$	87.65$\pm 7.43^2$	90.58$\pm 6.99^1$
Mushroom	JRip	92.68$\pm 2.71^3$	91.12$\pm 4.37^4$	93.89$\pm 2.21^2$	99.95$\pm 0.13^1$
	RF	95.44$\pm 1.51^3$	95.21$\pm 1.86^4$	97.28$\pm 1.09^2$	99.99$\pm 0.04^1$
qsarbio-degradation	JRip	84.80$\pm 3.87^{2.5}$	85.54$\pm 2.54^1$	82.21$\pm 2.89^4$	84.80$\pm 3.87^{2.5}$
	RF	89.45$\pm 3.23^3$	90.62$\pm 3.87^2$	86.29$\pm 2.98^4$	92.63$\pm 2.43^1$
Fertility diagonsis	JRip	75.59$\pm 10.01^{4}$	81.41$\pm 10.18^2$	83.17$\pm 9.99^1$	78.44$\pm 9.68^{3}$
	RF	81.43$\pm 9.73 ^4$	86.23$\pm 11.21^{2.5}$	86.23$\pm 11.21^{2.5}$	89.04$\pm 10.28^1$
Ionosphere	JRip	91.21$\pm 3.95^4$	95.55$\pm 3.78^{1.5}$	92.45$\pm 4.11^3$	95.55$\pm 3.78^{1.5}$
	RF	95.31$\pm 3.01^4$	98.12$\pm 2.67^{1.5}$	96.29$\pm 3.19^3$	98.12$\pm 2.67^{1.5}$
Thyroid-hypothyroid	JRip	92.41$\pm 2.19^3$	91.46$\pm 1.65^4$	98.22$\pm 1.05^2$	99.32$\pm 0.47^1$
	RF	95.41$\pm 2.01^3$	93.59$\pm 2.86^4$	99.11$\pm 0.98^2$	99.59$\pm 0.36^1$
wdbc	JRip	95.21$\pm 2.98^{3.5}$	95.21$\pm 2.98^{3.5}$	96.20$\pm 2.47^{1.5}$	96.20$\pm 2.47^{1.5}$
	RF	96.79$\pm 3.02^3$	96.28$\pm 1.86^4$	97.86$\pm 2.21^{1.5}$	97.86$\pm 2.21^{1.5}$
Average	JRip	3.50	2.60	2.35	1.55
Rank	RF	3.20	3.10	2.50	1.20
F statistics	JRip	5.68			
	RF	9.29			

Here, we have employed the three most prominent state-of-the-art techniques namely RBR, IFBR, and CFBR to perform the comparative performances. RBR struggles with information loss due to discretization process prior to apply the size and dimension reduction. This leads to degrade in the performance of the learning methods. Next, CFBR is an effective bireduct approach to handle the real-valued datasets, but it fails to handle the uncertainty due to both judgement and identification. This technique produces the reduced dataset, which usually guides the overfitting issue for complex data. IFBR technique can effectively handles the issues with RBR and CFBR, but it faces obstacles while handling overlapping available in the real-valued datasets. Moreover, IFBR usually takes more time to accomplish the reduction process. Our proposed method addresses all these obstacles by producing the reduced dataset for the real-valued datasets without performing discretization with the proposed similarity relation. IF set assisted idea helps in coping with uncertainty due to both judgement and identification. Covering concept avoids the overlapping issues during reduction process. The experimental results clearly declare the justification of theoretical proposal of the method. Tabular and pictorial results indicate that our proposed notion is usually providing effective results both in terms of data reduction and performance measures of the machine learning algorithms. Parameterized concepts in the proposed technique allows to discard the issues such redundancy, irrelevancy, and noise available in the large volume of high-dimensional datasets by adjusting the values of the parameters for the datasets from different domains.

### Case study: enhanced regression prediction model analysis for IC50 of antiviral peptides

Bioactive peptides known as antiviral peptides (AVPs) have virucidal action and show promise as antiviral medicines. They are peptides of shorter length with the capacity to stop the spread of viral infections. The research community has recently become interested in the medicinal application of antiviral peptides. For the development of new treatments for viral infections, AVP development and identification are essential. The prediction potency of AVPs measured in IC50 can provide valuable insights into the physichochemical space of high potency AVPs and facilitate in their rationale design. The proposed method is developed for selection of non-redundant features and informative samples for classification problems, but we further experimented with regression based modes IC50 value prediction. We first converted the AVP dataset consisting of IC50 values into a binary class dataset, followed by selection of informative instances and non-redundant features, then conversion of class labels with original IC50 values and further development of regression models on the bireduct dataset.

**Table 8:** Performance evaluation metrics for different learning algorithms for pIC50.

Algorithm	CC	MAE
GP-poly kernel	0.7101	0.7284
GP-PUK	0.8318	0.5539
GP-RBF	0.6452	0.8312
SMO-RBF	0.6863	0.7523
SMO-poly kernel	0.7289	0.6709
SMO-PUK	0.8599	0.3632
Decision table	0.7839	0.4977
Linear regression	0.7368	0.6837
IBK	0.7943	0.4116
RF	0.8577	0.4208
Vote (SMO-PUK+RF)	0.8652	0.3835
^81^	0.797	NA

**Table 9:** Performance evaluation metrics/parameters of different machine learning methods based on tenfold cross validation.

Learning methods	Sensitivity	Specificity	Accuracy	MCC	AUC
SMO	71.6	90.0	80.6	0.625	0.808
MLP	78.1	83.1	80.6	0.612	0.863
ROF	91.5	79.2	84.7	0.704	0.925
PART	84.4	80.8	82.6	0.652	0.848
J48	84.4	82.3	83.3	0.666	0.843
RF	89.7	83.8	86.7	0.736	0.950
RARF	90.9	85.6	88.2	0.766	0.956

**Figure 4:**
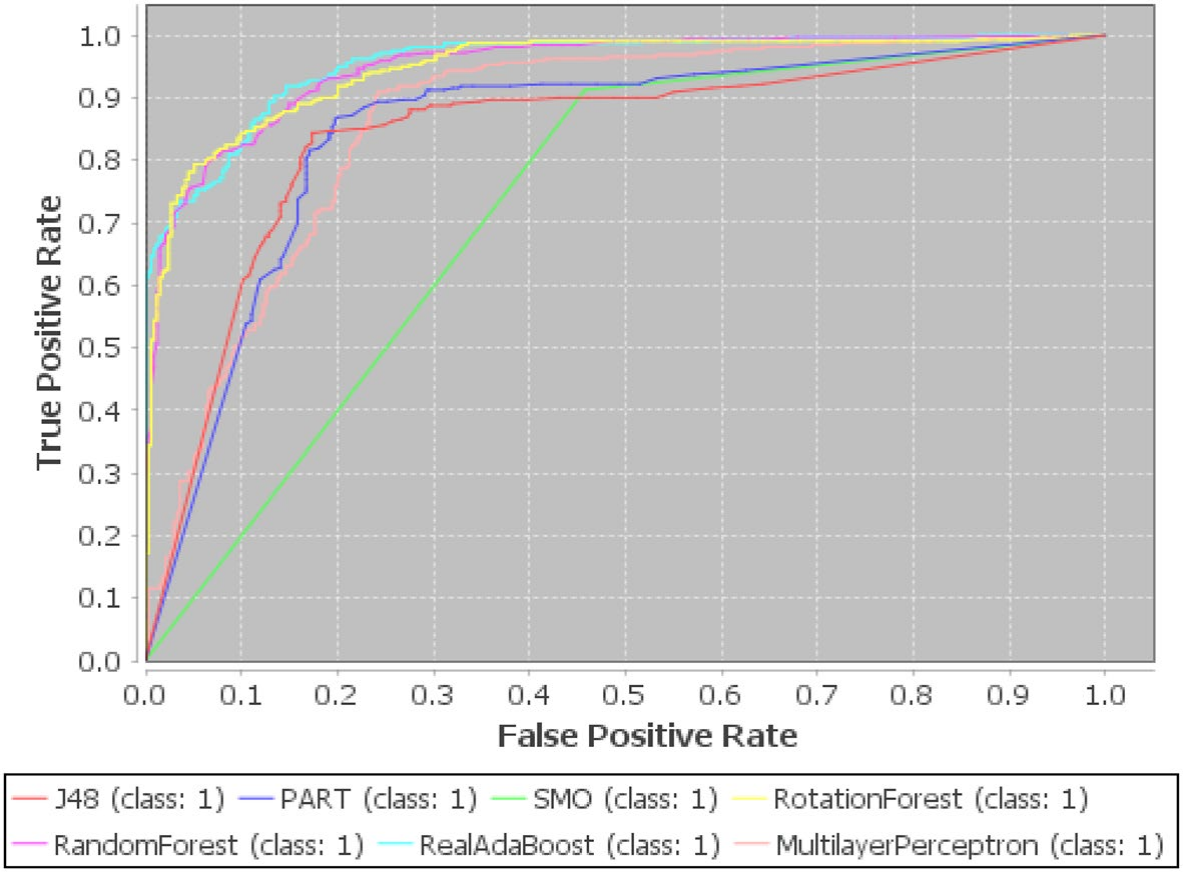
ROC curve for the visualization of performances of different learning algorithms for the binary dataset obtained from AVP dataset.

In Table 9, we have tabulated the classification results after converting the regression problem into binary classification problem by using median IC50 value as the cutoff for generating the binary classes for selection of non-redundant features and informative samples. The rationale for selecting median as the cutoff, is to divide the whole AVP dataset based on potency(measured in terms of IC50) and obtain a balanced dataset. The performance evaluation metrics for all the algorithms are similar. High sensitivity and accuracies values indicate that positive class prediction can be highly improved with the proposed methodology. AUC and MCC values are outlining that the proposed methodology is generating the relatively better predictor. In Table 8, the performance evaluation metrics for the different learning algorithms based on reduced datasets produced by our bi-selection method for the regression problem of predicting pIC50 values are tabulated. GP-PuK, SMO-PuK, RF, and Vote methods surpassed the previously reported best CC of 0.797. The best CC value is obtained by the vote method (CC = 0.8652) as given in Table 8. The dataset consisted of 683 AVPs with IC50 values, in line with the previous method^81^, where IC50 values are converted to pIC50 (negative log of half maximal inhibitory concentration) values, which facilitates in training as it compactly compresses the range of IC50 values. The features for the representation of AVPs consisted of SWScore (local alignment scores)which are effective for identifying patches of identical or similar amino acid residues that are conserved locally and regions of significant local similarity and evolutionary information in the form of PSSM (position specific scoring matrices) obtained from PSI-BLAST algorithm (parameters—e-value = 2000, word-size = 2, scoring matrix = PAM30, threshold = 16, gap open = 9, gap extend = 1, and word-size = 15). For better understanding of the different results, ROC curve is given by Fig. 4 to display the performances of machine learning methods. The original dataset for IC50/pIC50 prediction consisted of 449 length feature vector (each Antiviral protein is represented by 449 features, consisting of sequence and evolutionary information). In domains like AVPs IC50 value prediction, the use of lower prediction accuracy. The vote method consisting of support vector machine and random forest, achieved a value of 0.865 for correlation coefficient in comparison to original dataset where the vote with the same combination achieved 0.797. A rise of 7 points can be observed for the correlation coefficient metric (Table 8). This supports the utility of the proposed approach. The values of CC and MAE depict that our proposed methodology produces the reduced datasets by avoiding redundancy, uncertainty, and noise to enhance the regression performance better than the existing techniques.

**Table 10:** Performance evaluation metrics of different machine learning methods for reduced Arrhythmia dataset using 10-fold cross validation.

Learning methods	Precision	Recall	F-measure	Accuracy	AUC
SMO	84.2	79.5	81.8	79.5	0.826
Rotation forest	86.8	88.3	87.5	88.3	0.915
PART	81.0	81.3	81.2	81.3	0.858
J48	86.1	85.2	85.7	85.2	0.919
Vote	89.9	88.6	89.3	88.6	0.939
Random forest	87.2	86.0	86.6	86.0	0.940

**Table 11:** Performance evaluation metrics of different machine learning methods for reduced dataset consisted of antioxidant and non-antioxident peptides using tenfold cross validation.

Leaming methods	Precision	Recall	F-measure	Accuracy	AUC
SMO	74.3	72.4	73.3	72.4	0.741
Rotation forest	92.7	92.7	92.7	92.7	0.934
PART	88.3	88.3	88.3	88.3	0.899
J48	88.3	91.2	89.7	91.2	0.916
Vote	95.2	96.3	95.8	96.3	0.989
Random forest	93.7	92.5	93.1	92.5	0.962

Now, we conduct the experimental study in the field of signal processing and bioinformatics to validate the wider perspective of the proposed method as follows:

In the signal processing dataset^82^, the objective is to identify the various forms and manifestations of cardiac arrhythmia and assign it to one of the sixteen groups. There are currently 279 feature values that characterize 452 patient records. Class 01 pertains to normal ECG readings. Class 09 to left bundle branch block, class 10 to right bundle block, class 06 to sinus bradycardia, class 07 to ventricular premature contraction (PVC). Class 02 denotes ischemic alterations (coronary artery disease), class 03 denotes an old anterior myocardial infarction, class 04 denotes an old inferior myocardial infarction, and class 05 denotes sinus tachycardia. Class 11 corresponds to the first degree of AV block, Class 12 to the second degree, Class 13 to the third degree, Class 14 to left ventricule hypertrophy, Class 15 to atrial fibrillation or flutter, and Class 16 to the remaining cases.

In Table 10, the performance evaluation metrics for the various machine learning algorithms are tabulated for the reduced Arrhythmia dataset as produced by our presented models. A rise in all the performance evaluation metrics can be observed for all the machine learning algorithms using the reduced dataset. The best performance is achieved by Vote method reaching an accuracy of 88.6$\%$ on the reduced dataset (a rise of 18 points in comparison to original dataset). The best values are achieved as precision, recall, F-measure, and accuracy of 89.9$\%$, 88.6$\%$, 89.3$\%$, and 88.6$\%$ respectively.

The UniProt database was used to carefully select proteins with antioxidant activity for the dataset. Uncertain proteins, such as those with unusual letters like “B,” “X,” and “Z,” were excluded, and only proteins with antioxidant activity that had been experimentally verified were included. After this comprehensive screening, it was found that 710 protein sequences were the initial positive samples. For the negative samples, 1567 PDB proteins with identification values less than 20 $\%$ were chosen using the PISCES-culled approach. To cut down on redundancy and avoid homology bias, peptides that shared more than 60$\%$ of similar sequences were removed from the benchmark dataset using the CD-HIT program. Ultimately, 1805 protein sequences were added to the new dataset as negative samples^83^.

In Table 11, the performance evaluation metrics for the various machine learning algorithms are tabulated for the reduced dataset produced by our proposed models for discriminating the antioxidant and non-antioxident peptides. A rise in all the performance evaluation metrics can be observed for all the machine learning algorithms using the reduced dataset. The best performance is achieved by vote method reaching an overall accuracy of 96.3 $\%$ on the reduced dataset (a rise of 10 points in comparison to original dataset). An effective improvement in precision, recall, F-measure, and AUC is also reported based on our proposed method. The evaluation metrics values are guiding the conclusion that proposed methodology is producing the effective predictor for biological, signal processing, and bioinformatics data.

## Conclusion

The current study combined the notion of IF and rough sets for data reduction both in terms of instances and features. These reductions demonstrate that the reduced data can be produced whilst still maintaining the informative predictive facets, and hence avoid the complexity available in the large volume of real-valued datasets. An IF similarity relation was presented to establish IF similarity relation. Then, we investigated an IF granule based on this relation and thresholds. Relevant propositions were put forward to clarify the effectiveness of the proposed concepts theoretically. Next, instance selection algorithm was addressed by using importance degree of this granular structure, which considers representative instances by removing noise in the data. Thereafter, significance-degree-preserved feature selection idea was introduced, which was employed to eliminate redundant and irrelevant attributes. Further, the entire bireduct was applied over benchmark datasets to perform extensive experiments. From the comparative study with earlier discussed models clearly indicated the dominance of the presented bireduct method. Lastly, a methodology was highlighted as claimed by our proposed method to improve the regression analysis for IC50.

In the future, we intend to establish a new intuitionistic fuzzification of real-valued data to discard the possible information loss due to existing parameterized concept. Moreover, this method can be further improved by providing the generalized computation of the threshold parameters.

**Figure 5:**
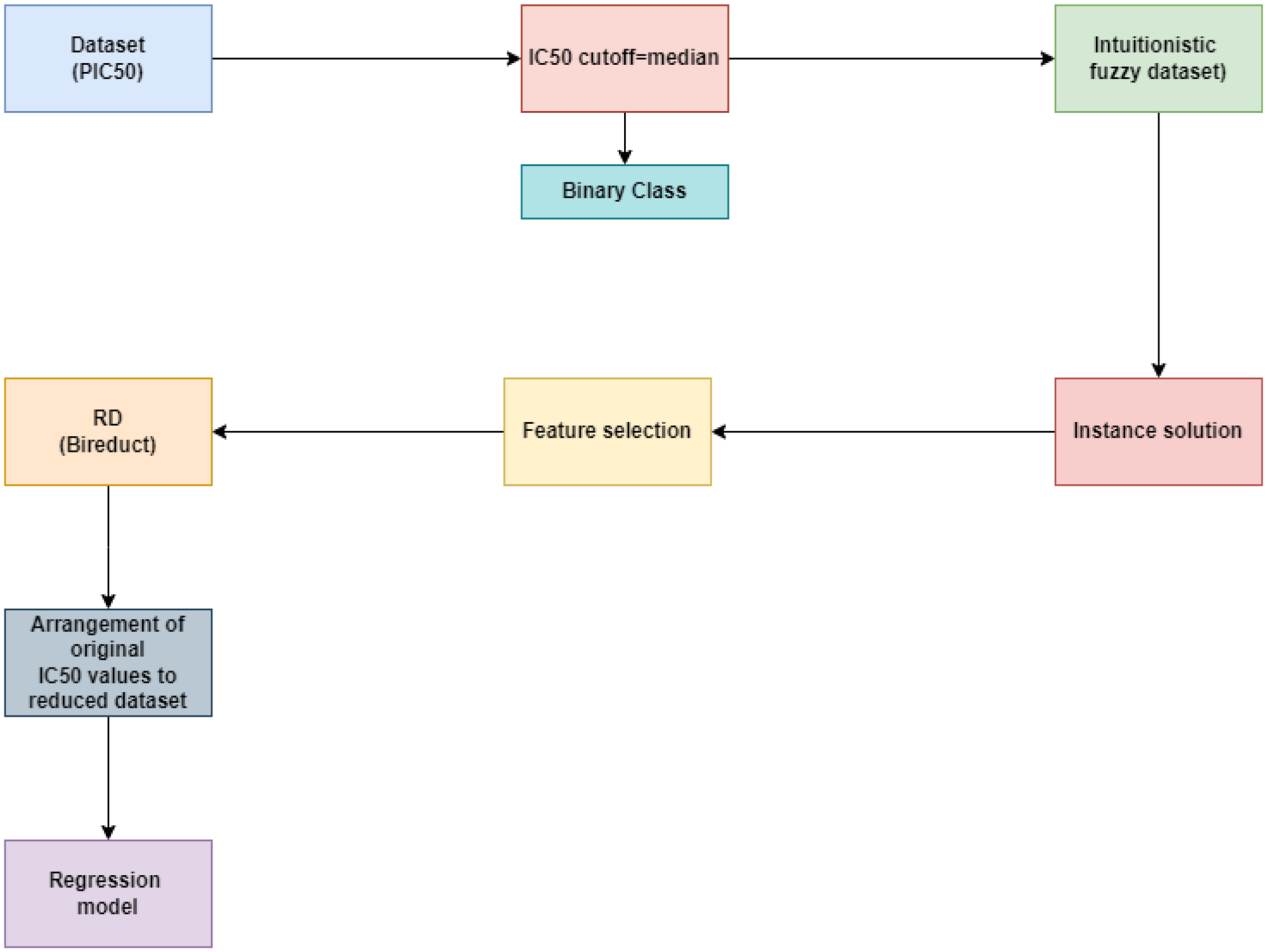
Schematic representation of the entire proposed methodology for regression analysis.

## Data Availability

The data supporting this study’s findings are available from the corresponding author (Mohd Asif Shah) upon reasonable request.
